# Multi-Omics Approaches to Study Long Non-coding RNA Function in Atherosclerosis

**DOI:** 10.3389/fcvm.2019.00009

**Published:** 2019-02-19

**Authors:** Adam W. Turner, Doris Wong, Mohammad Daud Khan, Caitlin N. Dreisbach, Meredith Palmore, Clint L. Miller

**Affiliations:** ^1^Center for Public Health Genomics, University of Virginia, Charlottesville, VA, United States; ^2^Department of Biochemistry and Molecular Genetics, University of Virginia, Charlottesville, VA, United States; ^3^School of Nursing, University of Virginia, Charlottesville, VA, United States; ^4^Data Science Institute, University of Virginia, Charlottesville, VA, United States; ^5^Department of Biomedical Engineering, University of Virginia, Charlottesville, VA, United States; ^6^Department of Public Health Sciences, University of Virginia, Charlottesville, VA, United States

**Keywords:** long noncoding (lnc) RNAs, genomics, cardiovascular disease, atherosclerosis, gene regulation

## Abstract

Atherosclerosis is a complex inflammatory disease of the vessel wall involving the interplay of multiple cell types including vascular smooth muscle cells, endothelial cells, and macrophages. Large-scale genome-wide association studies (GWAS) and the advancement of next generation sequencing technologies have rapidly expanded the number of long non-coding RNA (lncRNA) transcripts predicted to play critical roles in the pathogenesis of the disease. In this review, we highlight several lncRNAs whose functional role in atherosclerosis is well-documented through traditional biochemical approaches as well as those identified through RNA-sequencing and other high-throughput assays. We describe novel genomics approaches to study both evolutionarily conserved and divergent lncRNA functions and interactions with DNA, RNA, and proteins. We also highlight assays to resolve the complex spatial and temporal regulation of lncRNAs. Finally, we summarize the latest suite of computational tools designed to improve genomic and functional annotation of these transcripts in the human genome. Deep characterization of lncRNAs is fundamental to unravel coronary atherosclerosis and other cardiovascular diseases, as these regulatory molecules represent a new class of potential therapeutic targets and/or diagnostic markers to mitigate both genetic and environmental risk factors.

## Introduction

Despite intensive research into the underlying pathogenesis of atherosclerosis/coronary artery disease (CAD), this disease still remains a significant public health burden. Atherosclerosis is a complex disease involving both environmental and genetic risk factors resulting in plaque formation and inflammation in the vessel wall. A number of cellular responses have been proposed to contribute to disease progression, including endothelial cell dysfunction, vascular smooth muscle cell phenotypic switching, macrophage/foam cell activation/necrosis/defective efferocytosis, and defective lipid/lipoprotein metabolism (1, 2). Much of the research to date has focused on the role of protein coding genes in atherosclerosis leading to the identification of a number of proteins described as key drivers. However, our understanding of the causal mechanisms of this disease remains limited, likely due to our incomplete functional knowledge of the non-coding genome. It is now well-established that more than 90% of disease-associated variants reside in non-coding regions, once considered “junk sequences” (3). An increasing number of studies, especially those utilizing high-throughput sequencing technologies, have shown that a number of non-coding RNAs (ncRNAs) are differentially regulated during disease, supporting potential functional roles for these molecules (4).

The formation of the GENCODE project (part of the ENCODE project) and other large-scale initiatives such as NONCODE have revealed 75% of the genome is transcribed, yet only 2% encodes for protein, suggesting alternative functional roles for ncRNA transcripts (5, 6). Since the development of RNA-seq and other high-throughput sequencing assays, ncRNAs are now appreciated as key regulators of gene expression (7). A number of the essential types of non-coding RNAs are already well-characterized such as transfer, spliceosomal, and ribosomal RNAs. Aside from these housekeeping RNA elements, the remaining types of non-coding RNAs are subdivided into two classes, small and long non-coding RNAs, based on their size. Examples of small non-coding RNAs include microRNAs (miRNA), small nucleolar RNAs (snoRNA), and PIWI-interacting RNAs (piRNA) (8). These elements can act as positive or negative regulators of gene expression and generally exert their influence through complementary base pairing to their target transcript 3′ or 5′ untranslated regions (9).

Long non-coding RNAs (lncRNAs) represent a heterogeneous class of non-coding RNAs that includes transcripts >200 nucleotides, which lack functional protein coding ability (10). Within this lncRNA class, they are also classified based on their genomic location and broadly encompass enhancer-related RNAs (eRNAs), transcribed ultraconserved RNAs, intronic RNAs, long intergenic RNAs (lincRNAs), and natural antisense transcripts (NATs) (10). Contrary to canonical linear lncRNAs, a distinct group of lncRNAs are known as circular RNAs (circRNAs) due to their circular structure, which often results from backsplicing (11). By participating in both transcriptional and post-transcriptional stages, lncRNAs modulate gene expression through multiple distinct mechanisms. Further insight into these regulatory mechanisms will facilitate a better understanding of disease biology and identify additional viable targets for therapeutic intervention or diagnostics. Here, we present an overview of various lncRNAs relevant to atherosclerosis and highlight next-generation sequencing approaches to systematically investigate lncRNA function, as well as the ongoing challenges in this exciting field.

### Mechanisms of Long Non-coding RNA Function

LncRNAs are a heterogeneous class of ncRNAs (>200 nucleotides in length) that do not contain a functional open reading frame (12). LncRNAs can be encoded on either the sense or antisense DNA strand and may be located within a protein coding gene or in the intergenic regions (12). Similar to mRNAs, lncRNAs are transcribed by RNA polymerase II. Many transcripts are polyadenylated, multi-exonic, undergo RNA splicing, and contain a 5' cap. Often, their active promoters are marked with H3K4me3 and gene bodies have H3K36me3 histone modifications (13). Unlike protein coding genes, lncRNAs are not translated into protein and thereby lack functional initiation and termination codons (8). They are expressed at a much lower levels relative to their protein coding counterparts and lack robust evolutionary conservation. Despite this low level of conservation, the expression pattern of lncRNAs has been shown to be relatively cell/tissue specific (14–16).

The mechanism of action of these regulatory factors were categorized into four broad groups proposed by Wang and Chang (17). Signaling lncRNAs represent a class which exhibit a high degree of spatial and temporal specificity that serve a role in signal transduction. Once transcribed, these signaling lncRNAs have effector functions in activating appropriate downstream pathways in response to a stimulus. Additionally, their presence may indicate a particular developmental cell state, condition, or overall transcriptional activity (17). Another mechanism in which lncRNAs exert their regulatory function is by acting as decoy molecules to limit the availability of RNA binding factors to interact with their partners. By impairing the ability of chromatin remodelers, transcription factors, and miRNA from binding to their target genes, decoy lncRNAs can inhibit downstream effector functions (17). It is noteworthy that miRNAs also have the ability to target lncRNAs directly and thereby influence transcriptional regulation and vascular functions (18–20). Aided by their ability to bind protein as well as base pair with target sequences, guide lncRNAs are responsible for localizing transcriptional regulators to specific regions. Similar to the role of guide lncRNAs, scaffold lncRNAs use their protein binding ability to provide a surface to mediate protein-protein interactions (17). In these various ways, lncRNAs represent a distinct class of regulatory elements to modulate transcriptional activities.

## Overview of LncRNAs Involved in Atherosclerosis

### Functional Studies of LncRNAs in Atherosclerosis

**Figure 1:**
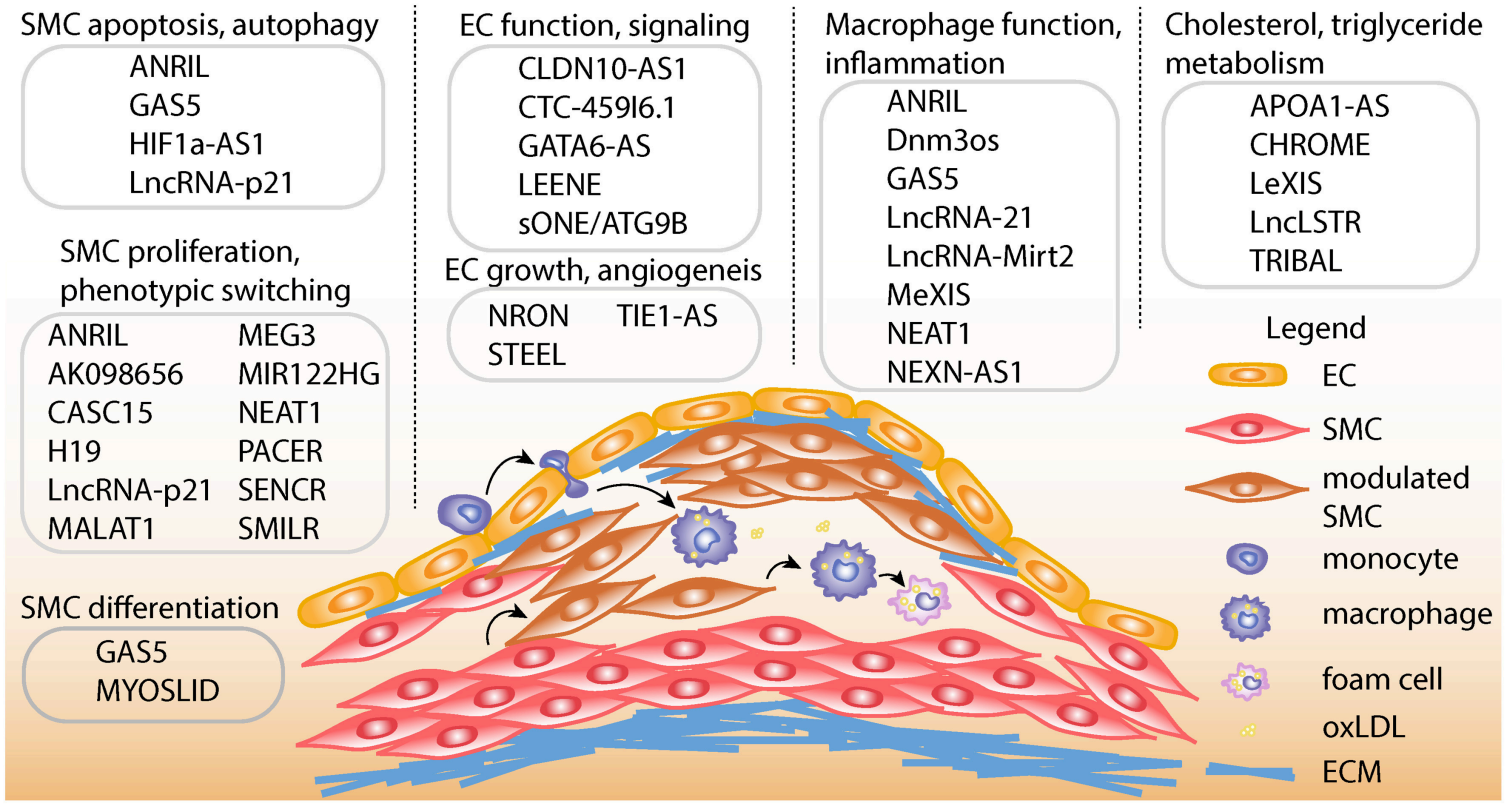
Schematic of atherosclerotic processes and specific lncRNA functions. Top, LncRNAs are shown with described smooth muscle cell (SMC) functions, such as proliferation, apoptosis, autophagy, phenotypic switching, and differentiation. LncRNAs are also shown with endothelial cell (EC) functions such as differentiation, regulation of endothelial nitric oxide synthase (eNOS) mediated signaling, growth and angiogenesis. LncRNAs are shown with macrophage functions, such as macrophage polarization, cholesterol efflux, and inflammation. Also, lncRNAs are listed with functions in regulating cholesterol and triglyceride metabolism in hepatocytes and/or macrophages. Bottom, schematic showing example of atherosclerotic lesion after invasion of vascular endothelium by activated monocytes, which become macrophages upon chronic inflammatory stimulation. Exposure to oxidized LDL (oxLDL) particles promote macrophage transformation to lipid-laden foam cells. Also depicted is the transformation of contractile SMCs to de-differentiated or modulated SMCs, as well as the transition of modulated SMCs to macrophage-like cells in the lesion. ECM, Extracellular matrix.

Depending on the cell types involved, lncRNAs play myriad roles in diverse atherosclerotic processes in the vessel wall including cell proliferation, migration, differentiation, apoptosis, and inflammation. They also play important roles in the regulation of cholesterol and lipid metabolism. Pertinent cell types include smooth muscle cells, endothelial cells, macrophages, and hepatocytes (Figure 1). A more comprehensive overview of key lncRNAs in atherogenic processes is given in Table 1. Perhaps the most well-studied in atherosclerosis is CDKN2B-AS1 or ANRIL (Antisense Non-coding RNA in the INK4 Locus), which acts in several cell types relevant to CAD (21, 22, 81). ANRIL acts as a guide lncRNA to localize polycomb repressive complex (PRC) at target promoters through a direct interaction with its subunits, CBX7 or SUZ12. PRC then adds H3K27me3 modifications to this region to repress transcription (22). Loss of function studies have suggested ANRIL acts in *cis* in order to regulate transcription of the nearby tumor suppressors, CDKN2A and CDKN2B (82, 83). Consistent with this mechanism of action, ANRIL expression correlates with a more proliferative phenotype in endothelial cells and vascular smooth muscle cells (VSMC) (22, 84, 85). In addition to acting in *cis*, ANRIL also acts in *trans* (via Alu elements) to regulate other genes that participate in proatherogenic pathways (22). Since ANRIL is not well-conserved in mice, *in vivo* functional studies have been challenging (86). A more complete overview of the atherogenic roles of ANRIL RNA species has been recently documented in a review by Holdt and Teupser (87).

**Table 1:** List of long non-coding RNAs with functional relevance in coronary artery disease cell types/tissues.

**Name**	**Full name**	**Initial detection method(s)**	**Cell types/tissues relevant to CAD**	**Function relevant to CAD**	**References**
ANRIL	A new large antisense non-coding RNA	Long range PCR and nucleotide sequencing	SMC, EC, Mac	Cell cycle regulation Regulation of apoptosis	(21–24)
GAS5	Growth arrest specific 5	Characterization of cDNA library	SMC, EC, Mac	Regulates apoptosis Regulates autophagy Regulates smooth muscle differentiation	(25–28)
HIF1a-AS1	Hypoxia-inducible factor 1-alpha Antisense RNA 1	RIP, qRT-PCR	SMC	Regulation of VSMC apoptosis	(29–32)
LincRNA-p21	Long intergenic non-coding RNA at p21 locus	Nimblegen lincRNA tiling microarray platform	SMC, Mac	Regulation of cell proliferation Regulation of apoptosis	(33, 34)
MALAT1	Metastasis associated lung adenocarcinoma transcript 1	Identified in numerous physiological processes	SMC, EC, Mac	Regulation of cell proliferation Regulation of inflammation Regulation of autophagy	(35–40)
MEG3	Maternally expressed 3	Characterization of cDNA library	EC, SMC	Regulates endothelial cell proliferation Inhibits angiogenesis Regulates smooth muscle proliferation/migration	(41–43)
NEAT1	Nuclear paraspeckle assembly transcript 1	Affymetrix expression array	SMC, Mac	Regulation of VSMC phenotypic switching Macrophage polarization	(44–47)
NEXN-AS1	Nexilin antisense transcript 1	lncRNA array	EC, SMC	Regulation of adhesion molecules/monocyte recruitment Regulation of inflammatory cytokines	(48)
CHROME	Cholesterol homeostasis regulator of miRNA expression	Characterization of transcript proximal to CAD and plasma HDL-C associated locus	Mac, Liver	Regulation of cholesterol homeostasis	(49)
AK098656	AK098656	lncRNA array	SMC	Regulation of VSMC phenotypic switching	(50)
CASC15	Cancer susceptibility 15	Quantification of copy number gains in metastatic melanoma	SMC	VSMC stiffness	(51, 52)
H19	H19	cDNA characterization	SMC	Regulation of VSMC differentiation Let 7 miRNA sponge	(53–55)
MIR122HG/ Lnc-Ang362	MicroRNA 122 Host Gene	RNA-seq	SMC	Regulation of VSMC proliferation	(56)
MYOSLID	MYOcardin-induced Smooth muscle LncRNA, Inducer of Differentiation	RNA-seq	SMC	Regulation of SMC differentiation	(57)
PACER	P50-associated COX-2 extragenic RNA	ANalysis of ChIP data, RT-qPCR	SMC	Regulation of COX-2 expression VSMC stiffness	(51, 58)
SENCR	Smooth muscle and Endothelial cell-enriched migration/differentiation-associated long Non-coding RNA	RNA-seq	SMC	Regulation of myocardin, SMC contractile gene program	(59)
SMILR	Smooth muscle-induced lncRNA enhances replication	RNA-seq	SMC	Regulation of VSMC proliferation	(60)
CLDN10-AS1	Claudin 10 antisense transcript 1	lncRNA microarray	EC	Regulation of endothelial signaling	(61)
CTC-459I6.1	RASGRF2 antisense RNA 1	lncRNA microarray	EC	Regulation of endothelial signaling	(61)
GATA6-AS	GATA6 antisense	RNA-seq	EC	Regulation of endothelial signaling Effects on endothelial- mesenchymal transition	(62)
LEENE	lncRNA that enhances eNOS expression	RNA-seq, chromosome conformation capture	EC	Regulation of eNOS and endothelial function	(63)
MIAT	Myocardial infarction associated transcript	Isolation of specific cDNA, RACE	EC	Regulation of angiogenesis	(64, 65)
NRON	Non-protein coding RNA, repressor of NFAT	Screen of cDNA library to identify conserved lncRNAsshRNA screen to identify NFAT regulators	EC	Regulation of angiogenesis Regulation of vascular development	(66–68)
sONE/ ATG9B	Autophagy related 9B	cDNA characterization	EC	Regulation of eNOS	(69)
STEEL	Spliced transcript endothelial-enriched lncRNA	Custom lncRNA microarray	EC	Angiogenesis	(70)
TIE1-AS	Endothelial-specific receptor tyrosine kinase 1 antisense	Detection in EST libraries, RACE	EC	Regulation of vascular development	(71, 72)
Dnm3os	Dynamin-3 opposite strand/antisense RNA	Isolation of clones, RACE	Mac	Regulation of macrophage inflammation	(73, 74)
lncRNA-Mirt2	lncRNA Myocardial infarction associated transcript	lncRNA expression microarray	Mac	Negative regulator of inflammation	(75)
MeXIS	Macrophage-expressed LXR-induced sequence	RNA-seq	Mac	Regulation of cholesterol metabolism	(76)
APOA1-AS	Apolipoprotein A1 antisense transcript	Identification in EST library, RACE	Liver	Regulation of APOA1 expression	(77)
LeXis	Liver-expressed LXR-induced sequence	RNA-seq	Liver	Regulation of cholesterol metabolism	(78)
lncLSTR	lncRNA Liver-Specific Triglyceride Regulator	Characterization of imprinted genes Reannotation of Affymetrix Mouse Genome 430 2.0 Array, search for liver-enriched transcripts	Liver	Regulation of triglyceride metabolism	(79)
TRIBAL	Tribbles homolog 1-associated locus	Identification in EST library, RACE	Liver	Regulation triglyceride metabolism	(80)

*Color scheme: Gray, lncRNAs associated with multiple cell types/tissues. Purple, lncRNAs associated with smooth muscle cells (SMC). Green, lncRNAs associated with endothelial cells (EC), Yellow, lncRNAs associated with macrophages (Mac), Blue, lncRNAs associated with cholesterol metabolism in liver. RACE: Rapid amplification of cDNA ends, EST: Expressed sequence tag*.

The ubiquitously expressed and evolutionarily conserved lncRNA, MALAT1, is decreased in atherosclerotic plaques (88, 89), and reduced MALAT1 levels in hematopoietic cells promotes atherosclerosis and inflammation in mice *in vivo* (89). In contrast, MALAT1 knockdown in VSMC and EC results in cell cycle arrest and reduced proliferation (35, 36). Other lncRNAs (e.g., lincRNA-p21, HIF1α-AS, circular ANRIL, and GAS5) have been implicated in cell death/apoptotic pathways through various mechanisms (23, 29, 33, 90), as described elsewhere (91, 92). In particular, lincRNA-p21 was shown to be reduced in CAD patients and mouse models of atherosclerosis, and regulates p53-dependent smooth muscle cell proliferation and apoptosis (33). Many lncRNAs have established immune and inflammatory roles. For example, heterozygous MALAT1-deficient ApoE-/- mice have increased inflammation and atherosclerosis (93). LncRNA Dnm3os (dynamin 3 opposite strand) is upregulated in diabetic induced macrophages and regulates nucleolin and epigenetic mediated inflammatory responses (73). Finally, in human macrophages treated with oxidized LDL, HOTAIR regulates oxidative stress and inflammation (94).

There are several lncRNAs with regulatory roles in lipid and cholesterol metabolism. CHROME (cholesterol homeostasis regulator of miRNA expression) is a lncRNA upregulated in carotid plaques, which regulates cholesterol homeostasis in primates in liver and macrophages by inhibiting miRNAs, such as miR-33 (49). NEAT1 promotes pro-atherogenic functions in THP-1 human macrophage cells such as increased ox-LDL lipid accumulation and inflammation by serving as a sponge of miR-342-3p target (44). Finally, differential expression of TRIBAL, APOA1-AS, and lncLSTR is linked to defects in lipid metabolic pathways, mainly in the liver. TRIBAL (TRIB1 associated locus) regulates Trib1 mRNA stability through mitogen activated kinase, consistent with Trib1 regulation (80, 95, 96). Increased TRIBAL expression stabilizes Trib1 expression and upregulates fatty acid oxidative pathways (80). Likewise, lncLSTR (liver-specific triglyceride regulator) regulates plasma triglyceride clearance by modulating apolipoprotein C2 (APOC2) levels and lipoprotein lipase activity (79). APOA1-AS regulates cholesterol levels through epigenetic modulation of APOA1, a protein involved in the cholesterol efflux pathway (77).

### LncRNAs With Genetic Associations in Atherosclerosis

Genome wide association studies (GWAS) have linked genetic variation at the ANRIL locus (9p21.3) to many complex phenotypes including CAD, stroke, type 2 diabetes and multiple cancers (97). In addition to ANRIL, the 9p21.3 locus encodes three tumor suppressor proteins: CDKN2A, CDKN2B, and MTAP. Despite each being attractive candidates underlying the locus association with various diseases, several studies report CAD risk polymorphisms associated with ANRIL expression (21, 98). However, association studies of 9p21.3 genotype with ANRIL expression remain complex due to the numerous linear and circular ANRIL forms (23).

Other less studied lncRNAs have been identified from genetic studies of CAD or related traits and may play critical roles in atherosclerosis. For instance, genetic variation in the imprinted lncRNA H19, involved in embryonic development (99) and oncogenesis (100), was associated with CAD and ischemic stroke in Chinese populations (101, 102). H19 was initially shown to be re-expressed in smooth muscle cells in human and rodent atherosclerotic plaques (103), and promotes VSMC proliferation by acting as a let-7a miRNA sponge to upregulate cyclin D (104). However, a recent study revealed endothelial cell restricted expression in human atherosclerotic plaques and a role in endothelial cell aging by suppressing STAT3 signaling (105), similar to lncRNA MEG3 (106). Another endothelial cell lncRNA, MIAT (Myocardial Infarction Associated Transcript), was previously associated with myocardial infarction in a large genetic study of a Japanese population (64). MIAT is upregulated in atherosclerosis plaques (88), and regulates microvascular dysfunction by acting as a competing endogenous RNA (65). Another lncRNA associated with CAD through large-scale GWAS is known as TARID (TCF21 antisense RNA inducing promoter demethylation) (107). TARID was identified as an eQTL target gene in human coronary artery smooth muscle cells (108), and molecular studies suggest this lncRNA guides GADD45A mediated DNA demethylation and inactivation of TCF21 (109), a known tumor suppressor and vascular wall transcription factor associated with CAD (110–113). Yet, functional studies of TARID both in VSMCs and *in vivo* are needed to elucidate its potential role in atherosclerosis. With larger GWAS sample sizes, and complementary eQTL colocalization (114), and transcriptome-wide association studies (TWAS) (115), it is anticipated that even more lncRNAs will be identified with genetic association evidence.

## Application of Transcriptomics to Identify and Study LncRNAs Relevant to CAD

### General Considerations for Transcriptomics Studies of LncRNAs

While traditional methods to profile lncRNA transcriptomes have relied on microarrays or serial analysis of gene expression (SAGE), these approaches have largely been replaced with the decreasing costs and greater output achieved by RNA-seq (116). In general, RNA-seq provides greater sensitivity and specificity to detect a broad range of ncRNA transcripts, novel isoforms, and interactions between ncRNAs (117). Nonetheless there are some important considerations when designing and conducting RNA-seq based lncRNA screening experiments. For instance, given that lncRNAs are approximately 10X less abundant than mRNAs on average, the basal expression of a typical lncRNA is < 5 fragments per kilobase of transcript per million mapped reads (FPKM) (118). Thus, it is highly recommended to obtain deeper sequencing per sample (~100X read depth) than a typical RNA-seq experiment. Also, while up to 50% of lncRNAs appear to be poly-adenylated (119) and would be detected with mRNA library preparation kits, a more comprehensive landscape of lncRNAs, other ncRNAs, including eRNAs, would require total RNA [poly(A) and non-poly(A)], ribosomal RNA depletion methods of purification. Distinguishing lncRNA transcripts from mRNA transcripts from short-read sequencing data remains a challenge, however deeper, paired-end and stranded sequencing should improve identification of lncRNAs (120). Also, careful study design is needed to ensure sufficient power to detect differentially expressed and transcript-specific lncRNAs, when using standard count-based tools (120). Since many lncRNAs are tissue and cell-specific (121–123), it is also worth considering the effects of diluting weak signals from bulk populations of cells, as well as specific environmental contexts that may regulate lncRNA transcript levels. Below, we summarize recent findings of RNA-seq based lncRNA discoveries in specific cell types relevant to CAD/atherosclerosis.

### Transcriptomics of Vascular Smooth Muscle Cell Function

The first VSMC lncRNA discovered via RNA-seq was Lnc-Ang362 (HG-MIR222), which is upregulated in rat aortic smooth muscle cells upon stimulation with angiotensin II (56). Lnc-Ang362 promotes VSMC proliferation and is the host-transcript for both *miR-221* and *miR-222*. Bell et al. conducted RNA-seq in human coronary artery smooth muscle cells and identified 31 previously unidentified lncRNAs (59). Notably, one of these was Smooth muscle and Endothelial cell-enriched migration/differentiation-associated long Non-coding RNA (SENCR), which is located antisense to the *FLI1* gene. SENCR functionally promotes a contractile smooth muscle phenotype and inhibits migration (59). In a follow-up study, RNA-seq was performed in human coronary artery smooth muscle cells to examine the effect of myocardin (MYOCD) overexpression (57). MYOCD is a potent co-factor that binds with serum response factor (SRF) to activate an array of smooth muscle-specific genes that maintain smooth muscle cell differentiation (124–128). Over 100 lncRNAs were differentially expressed, one of which was identified as MYOcardin-induced Smooth muscle LncRNA, Inducer of Differentiation (MYOSLID). Functional studies demonstrated that MYOSLID, a direct transcriptional target of MYOCD/SRF, promotes smooth muscle differentiation and inhibits proliferation (57).

Yu et al. used RNA-seq to compare transcriptomes of coronary and aortic smooth muscle cells subjected to both normal and pathological aortic stiffness, a subclinical risk factor for CAD and various aortic diseases (51). Only two of the top 20 ranked differentially expressed lncRNAs have been studied to date: CASC15 and PACER (RP5-973M2.2). These lncRNAs regulate expression of protein-coding genes in *cis* and PACER activates COX2 expression (52, 58, 129). Analysis of RNA-seq data highlighted the lncRNA MALAT1 as a key regulator of VSMC stiffness-induced proliferation and migration. Although MALAT1 was originally described as an endothelial lncRNA, MALAT1 regulates the phenotyping switching of VSMCs via activation of the autophagy pathway (36). Using RNA-seq in human smooth muscle cells Ballantyne et al. identified over 300 differentially expressed lncRNAs upon platelet-derived growth factor and interleukin-1 alpha stimulation. The novel lncRNA, Smooth Muscle-Induced LncRNA enhances Replication (SMILR) identified from this study enhances smooth muscle cell proliferation and has increased expression in unstable atherosclerotic plaques (60). The lncRNA *NEAT1* (nuclear paraspeckle assembly transcript 1) has recently been implicated in promoting the phenotypic switching of VSMCs (45). RNA-seq demonstrated *NEAT1* silencing increases the mRNA levels of numerous critical smooth muscle cell marker genes. Finally, to identify lncRNAs key in smooth muscle cell differentiation, Lim et al. combined and queried diverse RNA-seq datasets from Gene Expression Omnibus (GEO). Dozens of lncRNAs with no previous evidence for roles in VSMC differentiation were identified in this analysis that warrant further investigation, either as *cis* transcriptional regulators or suppressing miRNA function (130).

The development of custom lncRNA arrays has been applied to identify lncRNAs involved in various processes critical in atherosclerosis. One example is a microarray analysis which identified 580 lncRNAs differentially expressed upon exposure of human aortic smooth muscle cells to cyclic mechanical stretch (131). Another example is identification of AK098656, predominantly expressed in VSMCs, also upregulated in hypertensive patients and involved in promoting a synthetic smooth muscle cell phenotype (50).

### Transcriptomics of Endothelial Cell Function

Although not all lncRNAs have a poly(A) tail, Michalik et al. performed deep sequencing of poly(A)-selected RNA in human umbilical vein endothelial cells (HUVECs) and found over half of total RNA composed of non-coding RNA, many of which are lncRNAs (35). This study focused on five lncRNAs with high endothelial expression and strong conservation between mice and humans: MALAT1, linc00493, maternally expressed 3 (MEG3), taurine upregulated gene 1 (TUG1), and linc00657. MALAT1 and MEG3 are strongly upregulated in response to hypoxia while linc006757 are TUG1 are moderately upregulated. In regards to angiogenesis, MALAT1 promotes angiogenesis and induces a switch of endothelial cells from a migratory cell phenotype to a proliferative cell phenotype (132). Huang et al. postulated exosomal MALAT1 from oxidized LDL (oxLDL) treated endothelial cells (HUVECs) promotes macrophage polarization toward the M2 phenotype (133). MEG3 was shown to interact with epigenetic modifiers, to inhibit angiogenesis and contribute to age-related endothelial dysfunction (106, 134, 135).

In another study Miao et al. conducted RNA-seq profiling of endothelial cells subjected to both physiological and pathological flow for various time points (63). They identified and characterized LEENE (lncRNA that enhances eNOS expression) as a lncRNA highly correlated with endothelial nitric oxide synthase (eNOS) expression levels, which is downregulated upon pathological flow (63). Several lncRNAs characterized in smooth muscle cells also have functional significance in endothelial cells. For instance, the SMC lncRNA SENCR regulates the differentiation of pluripotent cells into endothelial cells and promotes angiogenesis in HUVECs (136).

Custom lncRNA microarrays have identified endothelial cell enriched lncRNAs (70) and endothelial lncRNAs differentially expressed in response to specific treatments (e.g., oxidized LDL) (61). These arrays have revealed new candidate lncRNAs in atherosclerosis including spliced-transcript endothelial-enriched lncRNA (STEEL) (70), NEXN-AS1 (48), CLDN10-AS1, and CTC-459I6.1 (61). STEEL facilitates the transcriptional stimulation of both eNOS and Kruppel-like factor (KLF2) and *in vivo* promotes angiogenesis (70). STEEL expression is decreased in HUVECs exposed to “atheroprotective” flow and expression is increased in HUVECs exposed to disturbed “atheroprone” flow.

### Transcriptomics of Macrophage Function and Inflammation

In macrophages, LXR activation promotes cholesterol efflux through activation of target genes such as *Abca1* during the formation of HDL. To investigate the regulation of LXR-dependent transcription in macrophages, a recent study conducted large-scale transcriptional profiling of mouse peritoneal macrophages in response to the LXR agonist GW3965. LXR activation stimulated transcription of an array of lncRNAs, of which MeXis was among the strongest induced (76). MeXis is well-conserved in mice and was shown to amplify the LXR-dependent expression of Abca1 *in vivo* and promote cholesterol efflux in macrophages (76). Loss of MeXis in Ldlr^−/−^ mice was shown to accelerate atherosclerosis through impaired Abca1 expression in macrophages and resulted in decreased cholesterol efflux (76). ATAC-seq in peritoneal macrophages demonstrated decreased chromatin accessibility across the *Abca1* locus in response to loss of MeXis. Querying the MeXis interactome through mass spectrometry revealed protein interactions with the nuclear receptor coactivator DDX17. Either directly or indirectly through one of its interacting targets, MeXis represents a potential therapeutic target to regulate macrophage cholesterol efflux.

RNA-seq and lncRNA arrays have identified a number of other macrophage lncRNAs that could represent novel CAD targets. Zhang et al. performed deep RNA sequencing of human monocyte-derived macrophages as well as M1 activated (via interferon gamma and lipopolysaccharide stimulation) and M2 activated (via interleukin 4 stimulation) macrophages (137). This study identified 861 previously unannotated lincRNAs, most of which are not syntenic in mouse. Furthermore, the lncRNA expression profile is dramatically shifted upon M1 activation, supporting the inflammatory nature of atherosclerosis. Similarly, 109 unannotated CD14^+^ monocyte lincRNAs were highlighted upon exposure to inflammatory stress *in vivo* (138). Other recent array studies highlighted the macrophage lncRNAs Dnm3os amd Mirt2. Dnm3os is upregulated in bone marrow derived macrophages in diabetic mice compared to controls and is higher in monocytes in human type 2 diabetic patients compared to controls (73). Dnm3os alters global histone modifications in macrophages and upregulates various immune-response and inflammatory genes. LncRNA-Mirt2 is strongly induced by LPS, a toll-like receptor 4 (TLR4) ligand where it acts as a negative feedback inflammatory regulator (75).

### Transcriptomics of Cholesterol Metabolism and Hepatocyte Function

Liver X receptors (LXRs) are nuclear factor transcription factors that are important mediators of lipid and cholesterol metabolism. LXR targets include the ABC family of transporters, ApoE, LPL, and SREBP (139, 140). Liver-specific LXR alpha knockout mice develop increased cholesterol levels and atherosclerosis (141). Sallam et al. performed genome-wide transcriptional profiling of primary mouse hepatocytes upon stimulation with an LXR agonist (78). The strongest induced gene was a non-coding RNA termed *LeXis* (liver-expressed LXR-induced sequence) that lies adjacent to the *Abca1* gene. LeXis regulates several genes with roles in cholesterol biosynthesis, subsequently altering both liver and plasma cholesterol levels. Mass spectrometry was used to characterize the LeXis interactome and revealed binding to RALY, a ribonucleoprotein that acts a transcriptional cofactor in regulation of cholesterol biosynthetic genes (78). In the context of atherosclerosis, adenoviral overexpression of LeXis in the liver reduces atherosclerosis in a familial hypercholesterolemia mouse model (142). As discussed above, CHROME is another LXR-regulated lncRNA involved in cholesterol homeostasis (49). CHROME was first identified through a combination of genetic association studies for premature CAD and HDL-C and microarray based expression profiling in human atherosclerotic plaques (49). RNA-seq of control and CHROME shRNA treated HepG2 hepatocytes revealed downstream pathways affected, including the LXR pathway, bile acid metabolism, cholesterol excretion and fatty-acid β-oxidation pathways (49).

## Application of Novel Genomic Technologies to Detect and Study LncRNA Functions

### Novel Sequencing Technologies to Discover and Annotate Long Non-coding RNAs

Although next-generation sequencing has resulted in the identification of thousands of lncRNAs in the genome, many of these lncRNAs remain poorly characterized and annotated. It is often unclear where transcription begins and which exons are present in a particular isoform. Since lncRNAs are often expressed at lower levels compared to protein-coding genes, current transcriptomic data is unable to provide comprehensive mapping/characterization of isoforms. However, new sequencing technologies allow for better characterization due to longer read lengths, higher sensitivity, and higher accuracy. Techniques such as Iso-Seq (Pacific Biosystems) offer long-read sequencing using single-molecule, real-time (SMRT) sequencing, in which the sequence of a full-length transcript is captured in a single read (143). Despite these benefits, these single-molecule sequencers yield higher error rates compared with short read sequencing technologies (e.g., Illumina). Nanopore technologies such as the MinION instrument (Oxford Nanopore Technologies) also allow single cDNA molecules to be sequenced without the need for amplification, providing sufficient read lengths to cover the full-length non-coding RNA, and results in less bias than other long-read approaches (144). This technique passes nucleic acids through an orifice 10^−9^ m in diameter, where instrumental electric current changes are utilized to decipher the identity of each nucleotide (145).

Since lncRNAs are typically less abundant than protein coding genes (usually one order of magnitude less), they remain a challenge to study in bulk transcriptomic datasets. To improve the detection and annotation of lncRNAs, a method known as RACE (Rapid Amplification of cDNA Ends)-Seq was developed (146), however this approach was limited by its low-throughput. Later a technique called RNA CaptureSeq was developed to enrich for long non-coding RNAs (147). RNA CaptureSeq employs an array of oligonucleotide probes to capture select genes of interest, which can be applied to pull-down lncRNAs of interest (148, 149). More recently the GENCODE consortium improved upon RNA CaptureSeq by developing RNA Capture Long Seq (RNA CLS) with the goal of annotating lncRNAs with much higher confidence (150). RNA CLS overcomes the short-read length hurdle of RNA CaptureSeq by first capturing lncRNAs and then integrating with long-read sequencing.

### DNA-Based LncRNA Interactions

**Figure 2:**
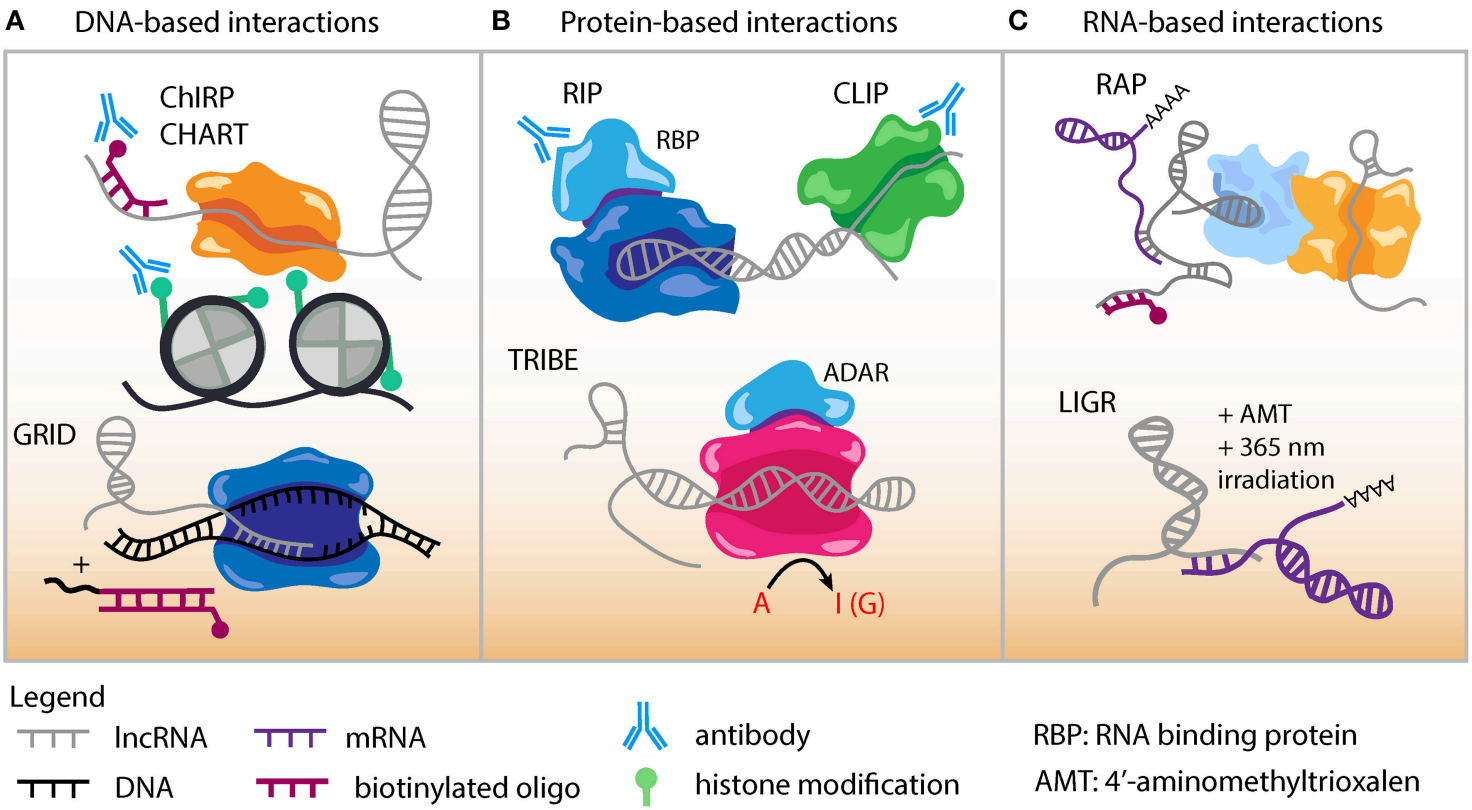
Genomic approaches to capture lncRNA interactions. **(A)** DNA-based lncRNA interactions include Chromatin Isolation by RNA Purification (ChIRP) and Capture Hybridization. Analysis of RNA Targets (CHART). An *in situ* based method to capture Global RNA Interactions with DNA (GRID) followed by deep sequencing uses a biotinylated bivalent linker to ligate RNA and dsDNA. **(B)** Protein-based lncRNA interactions include RNA Immunoprecipitation (RIP) which uses an antibody against RNA binding protein (RBP) to capture RNA-protein interactions. Cross-linking Immunoprecipitation (CLIP) combines UV cross-linking with immunoprecipitation to capture RNA-protein interactions. Targets of RNA-binding proteins Identified By Editing (TRIBE) couples an RBP to an RNA editing enzyme (ADAR). Targets of RBP are marked by adenosine to inositol RNA editing events and identified by sequencing. **(C)** RNA-based lncRNA interactions include RNA Antisense Purification, which uses a biotinylated probe to capture interacting RNAs that could be followed with sequencing or mass spectrometry. LIGation of interacting RNA (LIGR) followed by sequencing is a powerful approach to capture lncRNA-RNA interactions by *in vivo* crosslinking of RNA duplexes using the psoralen derivative 4'-aminomethyltrioxalen (AMT) and UV irradiation at 365 nm.

Despite their low abundance, lncRNAs are known to function through specific molecular interactions with other RNA species and RNA binding proteins. Several high-throughput methods are now available to uncover the genomic DNA sequences that lncRNAs interact with and likely regulate (Figure 2A). Chromatin Isolation by RNA Purification (ChIRP-Seq) is a well-established technique to study lncRNA-chromatin interactions through RNA/chromatin crosslinking, purification using biotinylated antisense oligonucleotides, followed by high-throughput sequencing (151, 152). Domain-specific ChIRP (dChIRP) is a variation of ChIRP that can characterize lncRNA function and architecture at the RNA domain level (153). dChIRP can not only investigate lncRNA-chromatin interactions but also pairwise lncRNA-RNA and lncRNA-protein interactions.

Capture hybridization analysis of RNA targets (CHART) (154, 155) is a similar method to experimentally determine where lncRNAs target and localize in the genome (Figure 2A). In the CHART protocol chromatin is crosslinked and lncRNAs subsequently hybridized to biotinylated C-oligos. After bead immobilization of lncRNA/DNA complexes, sequencing is conducted to identify lncRNA binding DNA regions.

GRID-seq (global RNA interactions with DNA by deep sequencing) is a new unbiased method to capture global RNA-interactions (Figure 2A) that can be applied to investigate lncRNA-DNA interactions in cell lines relevant to atherosclerosis (156). This GRID-seq technique uses a bivalent linker consisting of double-stranded DNA and single-stranded RNA to link RNAs with DNA in nuclei that have been fixed. Finally, MARGI (mapping RNA-genome interactions) is a high-throughput method that can be performed *in vivo* or on cells and reveal the genomic target sites of lncRNAs (157).

### Protein-Based LncRNA Interactions

ChIRP-MS is an adaptation of the ChIRP protocol and used to characterize the interacting proteome for a lncRNA (158). ChIRP-MS has identified protein interactors for lncRNAs such as LeXis, MeXis, and AK098656 (50, 76, 78). lncRNA pull-down followed by mass spectrometry has been conducted for several lncRNAs with potential roles in CAD such as circANRIL (23), STEEL (70), MALAT1 (159, 160), Dnm3os (73), lncLSTR (79), and GATA6-AS (62). Numerous additional methods exist to decipher the proteins binding lncRNAs. RAP-MS uses ultraviolet light to crosslink direct RNA-protein interactions (161). UV-C crosslinking immunoprecipitation (CLIP) is another powerful technique to interrogate direct protein-RNA interactions and many variations have been adapted based on the implementation of high-throughput sequencing (Figure 2B). These include iCLIP, PAR-CLIP, HITS-CLIP, irCLIP, and eCLIP (162–166). High-throughput sequencing of RNA isolation by crosslinking immunoprecipitation (HITS-CLIP) was developed as genome-wide means to interrogate RNA-protein interactions *in vivo* (164).

TRIBE (targets of RNA-binding proteins identified by editing) is designed for identifying RNA molecules that bind to RNA binding proteins (RBP) (Figure 2B) (167). Advantages of TRIBE include application to *in vivo* samples, ability to performed on a small number of cells, and no need for antibodies in the procedure. The TRIBE protocol couples an RNA editing enzyme to the RBP and RNA targets that have been edited are identified via next-generation sequencing (TRIBE-seq). HyperTRIBE extends upon the TRIBE procedure by introducing a hyperactive mutation into the RNA editing enzyme, which improves the RNA editing efficiency and reduces the sequence bias of editing (168).

### RNA-Based LncRNA Interactions

RNA-centric RNA antisense purification (RAP) is a general approach to identify and study lncRNA functions (Figure 2C). This method uses long capture probes (120 nucleotides) tiled across an entire RNA sequence to pull down lncRNAs, followed by stringent wash conditions to reduce non-specific binding (169). There are now next-generation sequencing derived methodologies that have been established to better define RNA-RNA interactions. LIGR-seq (LIGation of interacting RNA followed by high-throughput sequencing) can capture base-paired RNA-RNA interactions (Figure 2C) (170). In LIGR-seq, RNA duplexes are cross linked with the psoralen derivative 4'-aminomethyltrioxalen (AMT) along with UV irradiation at 365 nm, and RNase R is added to digest linear and structural RNAs. This step enriches for AMT-crosslinked RNA-RNA duplexes that are subsequently subjected to next-generation sequencing. Though LIGR-seq does not work well for small RNAs such as microRNA (miRNA), it should be able to uncover novel dynamic and long range interactions between lncRNAs and other RNA molecules. Various other methods have been developed to study the RNA interactome for lncRNAs with functional relevance in atherosclerosis including PARIS (Psoralen Analysis of RNA Interactions and Structures) (171), SPLASH (172), and MARIO (173). These techniques all can provide valuable information because many lncRNA sequence and structural motifs act as functional scaffolds in the assembly of RNA-protein complexes (17). However, it should be noted that many of these assays could be biased toward capturing stable interactions, while more transient and stimulation specific interactions may require some enrichment steps.

### *In situ* Hybridization-Based Methods

A critical consideration when interrogating a given lncRNA function, is identifying its endogenous tissue and cellular localization. While many lncRNAs are expected to be cytosolic and contribute to post-transcriptional, translational or post-translational gene regulation, nuclear lncRNAs could participate in transcriptional regulation, chromatin structure or mRNA export mechanisms (17). RNA Fluorescence *in situ* Hybridization (FISH) has been a traditional method to identify the subcellular localization of RNA within cells, however it lacks sensitivity for lowly expressed lncRNAs. Single-molecule RNA FISH (smFISH) is a quantitative technique that provides the sensitivity to detect these lncRNAs and measures absolute transcript levels by using multiple short probes per target RNA (174). However, given that smFISH relies heavily on the optical detection of a limited number of fluorophores, it is restricted in its multiplexing capacity. Attempts to overcome this issue include implementation of combinatorial labeling by spectral barcodes and the incorporation of sequential hybridizations (seqFISH) using different colored probes in each hybridization round (175, 176). In seqFISH individual transcripts are imaged as different colored dots and quantified by counting the number of dots. Multiplex error-robust combinatorial labeling (merFISH) is an *in situ* targeted approach that utilizes two-step labeling and the detection of binary barcodes assigned to specific targets. This is accomplished by several rounds of hybridization, imaging, and cleavage of fluorophores from probes conjugated to readout sequences that interchange each cycle. Hybridization to readout sequences by the merFISH technique is much less time consuming than methods that utilize hybridization directly to target RNAs (177, 178). RNA SPOTS (sequential probing of targets) follows the same rationale as merFISH, except that it is used *in vitro* instead of *in situ* (179).

While still in the nascent stage, the emergence of spatial transcriptomics facilitates integration of RNA-seq expression data with spatial locations of RNA molecules in individual tissue sections (180). In this procedure, fixed tissue samples are annealed to regionally barcoded reverse transcription primers. Following reverse transcription, RNA-seq followed by computational reconstruction allows the two-dimensional localization and quantification of RNA molecules (180). This barcoded method has already been applied to spatially resolve gene expression in the human adult heart (181). While this procedure was originally developed to study mRNAs, it shows promise for the spatial resolution of lncRNAs, given the increased sensitivity and ability to identify context-specific expression profiles. One consideration for atherosclerosis FISH experiments, is that heterogeneous cell types in lesions may be impacted differently by various fixation and hybridization conditions, so careful titration of reagents is recommended.

### Other LncRNA Functions

Finally, there are various omics methods that can define the dynamics of lncRNA transcription, stability and RNA modifications. Nascent RNA sequencing analysis, including global nuclear run-on sequencing (GRO-seq) and precision run-on sequencing (PRO-seq) assays, could enable comprehensive detection of transient RNA transcriptional events for multiple RNA species, including mRNA, lncRNA, and eRNA (182). While most transcriptomic datasets capture steady-state levels of lncRNA transcripts, they do not provide direct insights into the stability of lncRNAs. BRIC-seq (5'-bromo-uridine immunoprecipitation chase-deep sequencing analysis) is a method that pulse-labels endogenous RNAs and employs next-generation sequencing to measure RNA decay over time (183). Total RNAs (including lncRNAs) can be isolated from cells at desired time points under various cell-specific perturbations to facilitate functional analysis of lncRNA stability (e.g., lncRNA related to CAD). For example, direct measurements of lncRNA stability in response to CRISPR based loss/gain of gene function or drug treatments could be examined.

Another technique, ICE-seq (inosine chemical erasing coupled with sequencing) (184) represents a promising approach to globally identify lncRNA adenosine to inosine modifications (e.g., in the context of atherosclerosis). Adenosine to inosine (A-to-I) RNA editing is the most abundant form of RNA editing in humans and results from adenosine deaminase acting on RNA (ADAR). A-to-I editing is common to all lncRNAs and affects lncRNA function through altered stability and target recognition (185, 186). A-to-I RNA editing of mRNA has already been demonstrated to have important functional consequences in atherosclerosis. For example, A-to-I editing of cathepsin S mRNA (CTSS) is associated with cathepsin S levels in patients with atherosclerosis. Treatment of endothelial cells with inflammatory cytokines or exposure to hypoxia was shown to induce cathepsin S RNA editing and gene/protein expression (187).

## Novel Computational Tools for LncRNA Annotation and Functional Prediction

Genomic annotation of lncRNA sequences requires defining the precise genomic coordinates of lncRNA exons and their respective transcription start sites. LncRNA annotation also involves functional annotation with respect to predicted biological mechanisms, subcellular localization, and affected cell types/tissues. While lncRNAs share some similarities with mRNAs such as transcript length and splicing structure (188), proper identification and characterization of specific long non-coding transcripts still remains a challenge. Unlike mRNAs, lncRNAs often exhibit lower stability, lower abundance, less splicing and greater nuclear localization (189).

With the widespread application of high-throughput sequencing technologies, both automated and manual methods have been adopted to properly define lncRNA sequences from RNA-seq data. Automated annotation generates a larger catalog of lncRNAs and harnesses a transcriptome assembly consisting of two distinct strategies. In one automated approach, reads are first aligned to the reference genome to reveal all the possible splicing events which are subsequently assembled into transcripts (190, 191). In another automated approach, transcripts are built *de novo* from experimental reads and later aligned to a particular reference genome. Fu et al. (192) used both short and long sequencing reads to demonstrate superior sensitivity of transcript assembly and isoform annotation accuracy with the *de novo* approach. Automated assembly is fast as it does not require wet-lab based characterization, and it is considerable cheaper than the manual approach (144).

Although it produces a smaller catalog of lncRNAs compared to the automated method, manual annotation produces higher quality lncRNA transcript sequences and thus improves functional characterization. The widely adopted GENCODE project annotation of lncRNAs utilizes a manual curation approach (193), and integrates different sources of data together with computational analyses to generate a transcript model. cDNA and expressed sequence tag (EST) sequences deposited in publicly available databases are typically the starting point for manually annotating lncRNA transcripts. These are integrated with Cap Analysis of Gene Expression (5′-CAGE) and poly(A) position profiling by sequencing (3P-seq) to characterize 5′ and 3′ ends, respectively. These manually annotated transcripts are then mapped to reference genomes and assigned exon and splice site locations (119). The RefSeq (Reference Sequence) project also implements manual annotation of long non-coding RNAs that are integrated with automated methods (194). Manually annotated lncRNAs can be further divided into subclasses such as intergenic lncRNAs (lincRNAs), antisense lncRNAs, and intronic lncRNAs. As cDNA annotation depends on the availability of full length transcripts, manual annotation focuses primarily on genomic annotation. As a result the manual approach produces a more comprehensive set of pseudogenes and alternatively spliced transcripts (193).

Another comprehensive database established in 2016 is NONCODE that dedicates itself to collecting lncRNAs through integration with other databases (e.g., RefSeq and Ensembl) and exhaustive annotation. Compared to these other databases, NONCODE has collected more lncRNA transcripts (excluding tRNAs and rRNAs) and provides unique annotations of lncRNAs (e.g., RNA secondary structure, expression in exosomes, associations between lncRNA and disease) (6, 195). NONCODE also provides lncRNAs for over 15 species including mouse, zebrafish, and C-elegans. The latest version of NONCODE (v5), which also captures lncRNAs from the literature, consists of nearly 550,000 annotated lncRNAs (195).

There are now an array of computational tools to annotate the sequences and functions of the expanding catalog of lncRNAs, as described in Table 2. Existing computational methods for lncRNA identification include those that require a reference genome and those that are reference-free. Examples of methods requiring a reference genome include UClncR, lncScore (205), COME (206), and lncRScan-SVM (207). Reference-free methods to identify lncRNAs from RNA-seq data include LncADeep (198), lncRNAnet (197, 208), FEElnc (197), longdist (204), lncRNA-MFDL (209), and CPC2 (210). Many of these tools employ artificial intelligence algorithms (e.g., machine learning, deep learning) in order to distinguish lncRNAs from their protein-coding transcript counterparts.

**Table 2:** Comparison of selected computational tools.

Tool name and reference^*^	**Model**^**#**^	**Code available**	**Application**	**Performance results**
AnnoLnc (196)	Statistical approach	http://annolnc.cbi.pku.edu.cn	Annotation of human lncRNAs.	Not reported.
FEELnc (197)	Random forest	https://github.com/tderrien/FEELnc	Annotation of lncRNAs.	High classification power (AUC = 0.97).
LncADeep (198)	Deep belief network built as a stack of restricted Boltzmann machines	https://github.com/cyang235/LncADeep	Identification and functional annotation for lncRNAs	With 10-fold cross validation, average sensitivity of 98.1% and specificity of 97.2% and an average harmonic mean of 97.7%
LncFunTK (199)	Statistical approach	https://github.com/zhoujj2013/lncfuntk	To integrate ChIP-seq, CLIP-seq and RNA-seq data to predict, prioritize and annotate lncRNA functions.	Calculates a Functional Information Score (FIS) to quantitatively predict functional importance.
lncLocator (200)	Ensemble of support vector machine and random forest classifiers.	http://www.csbio.sjtu.edu.cn/bioinf/lncLocator/	To predict lncRNA subcellular localizations.	Accuracy of 59% for prediction.
PennDiff (201)	Regression-based statistical approach	https://github.com/tigerhu15/PennDiff	To detect differential transcript isoforms from RNA-seq data	Based on both annotations (RefSeq and Ensembl), estimates from PennDiff have Spearman correlation coefficients of 0.87 and 0.76, respectively.
SEEKR (202)	Statistical approach	https://github.com/CalabreseLab	Prediction of lncRNA subcellular localization, protein interactors	LncRNAs of related function have similar k-mer profiles, despite linear sequence similarity
UClncR (203)	Statistical approach	http://bioinformaticstools.mayo.edu/research/UClncR	Performs transcript assembly, prediction of lncRNA candidates in bulk RNA-seq data, quantification and annotation both known and novel lncRNA candidates.	For lincRNA prediction, UClncR reported 66 “novel” lincRNA transcripts and 12 lncRNAs overlapping with nearby genes (the recall rate of 90.7%).
A support vector machine based method to distinguish long non-coding RNAs from protein transcripts (204)	Support vector machine	https://github.com/hugowschneider/longdist.py	To distinguish lncRNAs from protein coding transcripts.	98.21% accuracy in classifying long non-coding RNAs from protein coding transcripts.

* *Three of the publications have not been constructed into available tools but rather represent a framework for analysis*.

# *Model type does not include preprocessing which may or may not including alignment of protein-coding regions. ^∧^The link is provided if the code is available otherwise the column is marked with an “X”. AUC, area under the curve*.

Unlike protein functions that can be inferred from protein-coding sequences, it is more difficult to infer lncRNA function from RNA sequences. Zhou et al. developed a tool, lncFunTK that calculates a Functional Information Score (FIS) to quantitatively measure the functional importance of a lncRNA (199), based on the top Gene Ontology and inferred regulatory networks for lncRNAs and their neighboring genes. Another tool, FEELnc, annotates lncRNA function by evaluating neighboring genes to predict both lncRNA function and mRNA partners (197). Given that lncRNA function often depends on subcellular localization, the lncLocator tool predicts five lncRNA categories: nucleus, cytoplasm, cytosol, exosome, and ribosome (200). LncADeep provides enriched pathways and functional modules for lncRNA functional annotation by integrating KEGG and Reactome Pathway databases in a deep learning framework (211). A novel method for lncRNA classification is SEEKR, which counts lncRNA k-mer frequencies from nucleotide sequences, which may be correlated with lncRNA localization or protein binding (202).

Like other ncRNAs, lncRNA functional annotation also depends on accurate secondary structure prediction. There are several computational tools to predict lncRNA structures such as pysster (212), RNAfold (213), RNAstructure (214), and UNAfold (215). Other tools are available to study the evolutionary conservation of lncRNAs, including EvoFold (216), Evolinc (217), RNAz (218), and SISSIz (219). Since lncRNAs are generally poorly conserved, there are strategies to examine remnants of protein coding sequences in these lncRNAs (220). Identifying evolutionarily conserved lncRNA structures and binding domains may provide clues to predict lncRNA function for follow-up experimentation.

## Conclusion

The emergence of RNA-seq and other omics technologies in the past decade have catalyzed the identification of a plethora of novel lncRNAs. To date, more than 30 lncRNAs with functional relevance to CAD have been characterized (Table 1), yet numerous lncRNAs remain to be studied in greater detail that are linked to endothelial, smooth muscle, macrophage, and lipid traits. With the growing number of CAD GWAS candidate loci harboring lncRNAs, and improved fine-mapping and annotation approaches, there is an opportunity to functionally dissect these regions to develop novel strategies to target non-coding genomic risk factors. As outlined in this review, a multi-faceted approach is likely required to successfully prioritize and study these lncRNAs, which may include implementation of long-read and high-depth sequencing, improved computational tools, coupled with orthogonal high-throughput experimental validation assays. Careful consideration of the lower abundance, context-specific expression of lncRNAs, and thoughtful study designs may improve chances of success in these multi-omics assays. However, it should also be noted that in many cases, more traditional and lower throughput approaches would be equally appropriate to characterize a given lncRNA, thus reducing the overall costs and required expertise.

For conserved lncRNAs with predicted roles in altering CAD pathogenesis, loss of function studies can be performed in animal models, such as the mouse (ApoE^−/−^ or LDLR^−/−^ backgrounds) or zebrafish. However, with the majority of human lncRNAs being poorly conserved across species, they may be better suited to studies in primary human cells or induced pluripotent stem cell (iPSC) derived vascular cells. In the context of CAD and other cardiometabolic disorders, genetic manipulation of lncRNAs via antisense oligonucleotides (221) or CRISPR/Cas9 to either delete (23, 222, 223) or activate/repress lncRNA expression, may lead to the identification of specific lncRNA binding partners, subcellular localization and functional insights relevant to CAD. lncRNA discovery/annotation can be further improved by integrating these genetic perturbations with high-dimensional transcriptomic and epigenomic assays (e.g., RNA-seq, ATAC-seq and ChIP-seq) to mark lncRNA promoters, decipher RNA polymerase and transcription factor binding, and reveal the dynamics of lncRNA regulatory activities. Single-cell based assays may also shed light on cell-specific markers and dynamics of lncRNAs across lineages (224, 225). Unraveling the complexity of lncRNA function in the setting of atherosclerosis may hold the key to delineate causal disease-associated pathways. In this regard it will also be important to determine whether lncRNAs operate synergistically, serve redundant and/or compensatory roles with other dysregulated lncRNAs and/or mRNAs associated with CAD.

## Author Contributions

AT and CM conceived of the manuscript. AT, DW, MK, CD, MP, and CM wrote the manuscript.

### Conflict of Interest Statement

The authors declare that the research was conducted in the absence of any commercial or financial relationships that could be construed as a potential conflict of interest.

## References

[1] Weber C Noels H. Atherosclerosis: current pathogenesis and therapeutic options. Nat Med. (2011) 17:1410–22. 10.1038/nm.2538 22064431

[2] Tabas I García-Cardeña G Owens GK. Recent insights into the cellular biology of atherosclerosis. J Cell Biol. (2015) 209:13–22. 10.1083/jcb.201412052 25869663 PMC4395483

[3] St. Laurent G Vyatkin Y Kapranov P. Dark matter RNA illuminates the puzzle of genome-wide association studies. BMC Med. (2014) 12:97. 10.1186/1741-7015-12-97 24924000 PMC4054906

[4] Liu Y Zheng L Wang Q Hu Y-W. Emerging roles and mechanisms of long noncoding RNAs in atherosclerosis. Int J Cardiol. (2017) 228:570–82. 10.1016/j.ijcard.2016.11.182 27875736

[5] Djebali S Davis CA Merkel A Dobin A Lassmann T Mortazavi A. Landscape of transcription in human cells. Nature (2012) 489:101–8. 10.1038/nature11233 22955620 PMC3684276

[6] Zhao Y Li H Fang S Kang Y Wu W Hao Y. NONCODE 2016: an informative and valuable data source of long non-coding RNAs. Nucleic Acids Res. (2016) 44:D203–8. 10.1093/nar/gkv1252 26586799 PMC4702886

[7] Palazzo AF Lee ES. Non-coding RNA: what is functional and what is junk? Front Genet. (2015) 6:2. 10.3389/fgene.2015.00002 25674102 PMC4306305

[8] Bhartiya D Scaria V. Genomic variations in non-coding RNAs: Structure, function and regulation. Genomics (2016) 107:59–68. 10.1016/j.ygeno.2016.01.005 26790601

[9] Costa MC Leitão AL Enguita FJ. Biogenesis and mechanism of action of small non-coding RNAs: insights from the point of view of structural biology. Int J Mol Sci. (2012) 13:10268–95. 10.3390/ijms130810268 22949860 PMC3431858

[10] Bhat SA Ahmad SM Mumtaz PT Malik AA Dar MA Urwat U. Long non-coding RNAs: mechanism of action and functional utility. Noncoding RNA Res. (2016) 1:43–50. 10.1016/j.ncrna.2016.11.002 30159410 PMC6096411

[11] Bartsch D Zirkel A Kurian L. Characterization of Circular RNAs (circRNA) Associated with the Translation Machinery. Methods Mol Biol. (2018). 1724:159–66. 10.1007/978-1-4939-7562-4_13 29322448

[12] Shi X Sun M Liu H Yao Y Song Y. Long non-coding RNAs: a new frontier in the study of human diseases. Cancer Lett. (2013) 339:159–66. 10.1016/j.canlet.2013.06.013 23791884

[13] Guttman M Amit I Garber M French C Lin MF Feldser D. Chromatin signature reveals over a thousand highly conserved large non-coding RNAs in mammals. Nature (2009) 458:223–7. 10.1038/nature07672 19182780 PMC2754849

[14] Pang KC Frith MC Mattick JS Rapid evolution of noncoding RNAs: lack of conservation does not mean lack of function. Trends Genet. (2006) 22:1–5. 10.1016/j.tig.2005.10.003 16290135

[15] Qu Z Adelson DL. Evolutionary conservation and functional roles of ncRNA. Front Genet. (2012) 3:205. 10.3389/fgene.2012.00205 23087702 PMC3466565

[16] Diederichs S. The four dimensions of noncoding RNA conservation. Trends Genet. (2014) 30:121–3. 10.1016/j.tig.2014.01.004 24613441

[17] Wang KC Chang HY. Molecular mechanisms of long noncoding RNAs. Mol Cell. (2011) 43:904–14. 10.1016/j.molcel.2011.08.018 21925379 PMC3199020

[18] Natarelli L Geißler C Csaba G Wei Y Zhu M di Francesco A. miR-103 promotes endothelial maladaptation by targeting lncWDR59. Nat Commun. (2018) 9:2645. 10.1038/s41467-018-05065-z 29980665 PMC6035258

[19] Santovito D Egea V Weber C. Small but smart: MicroRNAs orchestrate atherosclerosis development and progression. Biochim Biophys Acta (2016) 1861(12 Pt B):2075–86. 10.1016/j.bbalip.2015.12.013 26738655

[20] Feinberg MW Moore KJ. Microrna regulation of atherosclerosis. Circ Res. (2016) 118:703–20. 10.1161/CIRCRESAHA.115.306300 26892968 PMC4762069

[21] Jarinova O Stewart AFR Roberts R Wells G Lau P Naing T. Functional analysis of the chromosome 9p21.3 coronary artery disease risk locus. Arterioscler Thromb Vasc Biol. (2009) 29:1671–7. 10.1161/ATVBAHA.109.189522 19592466

[22] Holdt LM Hoffmann S Sass K Langenberger D Scholz M Krohn K. Alu elements in ANRIL non-coding RNA at chromosome 9p21 modulate atherogenic cell functions through trans-regulation of gene networks. PLoS Genet. (2013) 9:e1003588. 10.1371/journal.pgen.1003588 23861667 PMC3701717

[23] Holdt LM Stahringer A Sass K Pichler G Kulak NA Wilfert W. Circular non-coding RNA ANRIL modulates ribosomal RNA maturation and atherosclerosis in humans. Nat Commun. (2016) 7:12429. 10.1038/ncomms12429 27539542 PMC4992165

[24] Pasmant E Laurendeau I Héron D Vidaud M Vidaud D Bièche I. Characterization of a germ-line deletion, including the entire INK4/ARF locus, in a melanoma-neural system tumor family: identification of ANRIL, an antisense noncoding RNA whose expression coclusters with ARF. Cancer Res. (2007) 67:3963–9. 10.1158/0008-5472.CAN-06-2004 17440112

[25] Schneider C King RM Philipson L. Genes specifically expressed at growth arrest of mammalian cells. Cell (1988) 54:787–93. 10.1016/S0092-8674(88)91065-3 3409319

[26] Coccia EM Cicala C Charlesworth A Ciccarelli C Rossi GB Philipson L. Regulation and expression of a growth arrest-specific gene (gas5) during growth, differentiation, and development. Mol Cell Biol. (1992) 12:3514–21. 10.1128/MCB.12.8.3514 1630459 PMC364604

[27] Chen L Yang W Guo Y Chen W Zheng P Zeng J. Exosomal lncRNA GAS5 regulates the apoptosis of macrophages and vascular endothelial cells in atherosclerosis. PLoS ONE (2017) 12:e0185406. 10.1371/journal.pone.0185406 28945793 PMC5612752

[28] Tang R Zhang G Wang Y-C Mei X Chen S-Y. The long non-coding RNA GAS5 regulates transforming growth factor β (TGF-β)-induced smooth muscle cell differentiation via RNA Smad-binding elements. J Biol Chem. (2017) 292:14270–8. 10.1074/jbc.M117.790030 28659340 PMC5572929

[29] Wang S Zhang X Yuan Y Tan M Zhang L Xue X. BRG1 expression is increased in thoracic aortic aneurysms and regulates proliferation and apoptosis of vascular smooth muscle cells through the long non-coding RNA HIF1A-AS1 *in vitro*. Eur J Cardiothorac Surg. (2015) 47:439–46. 10.1093/ejcts/ezu215 24875884

[30] Baranello L Bertozzi D Fogli MV Pommier Y Capranico G. DNA topoisomerase I inhibition by camptothecin induces escape of RNA polymerase II from promoter-proximal pause site, antisense transcription and histone acetylation at the human HIF-1alpha gene locus. Nucleic Acids Res. (2010) 38:159–71. 10.1093/nar/gkp817 19854946 PMC2800211

[31] Bertozzi D Iurlaro R Sordet O Marinello J Zaffaroni N Capranico G. Characterization of novel antisense HIF-1α transcripts in human cancers. Cell Cycle (2011) 10:3189–97. 10.4161/cc.10.18.17183 21897117

[32] Zhao Y Feng G Wang Y Yue Y Zhao W. Regulation of apoptosis by long non-coding RNA HIF1A-AS1 in VSMCs: implications for TAA pathogenesis. Int J Clin Exp Pathol. (2014) 7:7643–52. 25550800 PMC4270571

[33] Wu G Cai J Han Y Chen J Huang Z-P Chen C. LincRNA-p21 regulates neointima formation, vascular smooth muscle cell proliferation, apoptosis, and atherosclerosis by enhancing p53 activity. Circulation (2014) 130:1452–65. 10.1161/CIRCULATIONAHA.114.011675 25156994 PMC4244705

[34] Huarte M Guttman M Feldser D Garber M Koziol MJ Kenzelmann-Broz D. A large intergenic noncoding RNA induced by p53 mediates global gene repression in the p53 response. Cell (2010) 142:409–19. 10.1016/j.cell.2010.06.040 20673990 PMC2956184

[35] Michalik KM You X Manavski Y Doddaballapur A Zörnig M Braun T. Long noncoding RNA MALAT1 regulates endothelial cell function and vessel growth. Circ Res. (2014) 114:1389–97. 10.1161/CIRCRESAHA.114.303265 24602777

[36] Song T-F Huang L-W Yuan Y Wang H-Q He H-P Ma W-J. LncRNA MALAT1 regulates smooth muscle cell phenotype switch via activation of autophagy. Oncotarget. (2018) 9:4411–26. 10.18632/oncotarget.23230 29435112 PMC5796983

[37] Luo J-H Ren B Keryanov S Tseng GC Rao UNM Monga SP. Transcriptomic and genomic analysis of human hepatocellular carcinomas and hepatoblastomas. Hepatology (2006) 44:1012–24. 10.1002/hep.21328 17006932 PMC1769554

[38] Guo F Li Y Liu Y Wang J Li Y Li G. Inhibition of metastasis-associated lung adenocarcinoma transcript 1 in CaSki human cervical cancer cells suppresses cell proliferation and invasion. Acta Biochim Biophys Sin (Shanghai) (2010) 42:224–9. 10.1093/abbs/gmq008 20213048

[39] Koshimizu T Fujiwara Y Sakai N Shibata K Tsuchiya H. Oxytocin stimulates expression of a noncoding RNA tumor marker in a human neuroblastoma cell line. Life Sci. (2010) 86(11–12):455–60. 10.1016/j.lfs.2010.02.001 20149803

[40] Guffanti A Iacono M Pelucchi P Kim N Soldà G Croft LJ. A transcriptional sketch of a primary human breast cancer by 454 deep sequencing. BMC Genomics (2009) 10:163. 10.1186/1471-2164-10-163 19379481 PMC2678161

[41] Miyoshi N Wagatsuma H Wakana S Shiroishi T Nomura M Aisaka K. Identification of an imprinted gene, Meg3/Gtl2 and its human homologue MEG3, first mapped on mouse distal chromosome 12 and human chromosome 14q. Genes Cells (2000) 5:211–20. 10.1046/j.1365-2443.2000.00320.x 10759892

[42] Wu Z He Y Li D Fang X Shang T Zhang H. Long noncoding RNA MEG3 suppressed endothelial cell proliferation and migration through regulating miR-21. Am J Transl Res. (2017) 9:3326–35. 28804550 PMC5553882

[43] Zhu B Gong Y Yan G Wang D Qiao Y Wang Q. Down-regulation of lncRNA MEG3 promotes hypoxia-induced human pulmonary artery smooth muscle cell proliferation and migration via repressing PTEN by sponging miR-21. Biochem Biophys Res Commun. (2018) 495:2125–32. 10.1016/j.bbrc.2017.11.185 29198701

[44] Wang L Xia J-W Ke Z-P Zhang B-H Blockade of NEAT1 represses inflammation response and lipid uptake via modulating miR-342–3 p in human macrophages THP-1 cells. J Cell Physiol. (2019) 234:5319–26. 10.1002/jcp.27340 30259979

[45] Ahmed ASI Dong K Liu J Wen T Yu L Xu F. Long noncoding RNA NEAT1 (nuclear paraspeckle assembly transcript 1) is critical for phenotypic switching of vascular smooth muscle cells. Proc Natl Acad Sci USA. (2018) 115:E8660–7. 10.1073/pnas.1803725115 30139920 PMC6140535

[46] Hutchinson JN Ensminger AW Clemson CM Lynch CR Lawrence JB Chess A. A screen for nuclear transcripts identifies two linked noncoding RNAs associated with SC35 splicing domains. BMC Genomics (2007) 8:39. 10.1186/1471-2164-8-39 17270048 PMC1800850

[47] Imamura K Imamachi N Akizuki G Kumakura M Kawaguchi A Nagata K. Long noncoding RNA NEAT1-dependent SFPQ relocation from promoter region to paraspeckle mediates IL8 expression upon immune stimuli. Mol Cell (2014) 53:393–406. 10.1016/j.molcel.2014.06.013 24507715

[48] Hu Y-W Guo F-X Xu Y-J Li P Lu Z-F McVey DG Long non-coding RNA NEXN-AS1 mitigates atherosclerosis by regulating the actin-binding protein NEXN. J Clin Invest. (2018). 10.1172/JCI98230 [Epub ahead of print]. PMC6391138 30589415

[49] Hennessy EJ van Solingen C Scacalossi KR Ouimet M Afonso MS Prins J The long noncoding RNA CHROME regulates cholesterol homeostasis in primates. Nat Metab. (2018). 1:98–110. 10.1038/s42255-018-0004-9 PMC6691505 31410392

[50] Jin L Lin X Yang L Fan X Wang W Li S. AK098656, a novel vascular smooth muscle cell-dominant long noncoding RNA, promotes hypertension. Hypertension (2018) 71:262–72. 10.1161/HYPERTENSIONAHA.117.09651 29279317

[51] Yu CK Xu T Assoian RK Rader DJ. Mining the stiffness-sensitive transcriptome in human vascular smooth muscle cells identifies long noncoding RNA stiffness regulators. Arterioscler Thromb Vasc Biol. (2018) 38:164–73. 10.1161/ATVBAHA.117.310237 29051139 PMC5881588

[52] Lessard L Liu M Marzese DM Wang H Chong K Kawas N. The CASC15 long intergenic noncoding RNA locus is involved in melanoma progression and phenotype switching. J Invest Dermatol. (2015) 135:2464–74. 10.1038/jid.2015.200 26016895 PMC4567947

[53] Feil R Walter J Allen ND Reik W. Developmental control of allelic methylation in the imprinted mouse Igf2 and H19 genes. Development (1994) 120:2933–43. 7607083 10.1242/dev.120.10.2933

[54] Feinberg AP Kalikin LM Johnson LA Thompson JS. Loss of imprinting in human cancer. Cold Spring Harb Symp Quant Biol. (1994) 59:357–64. 10.1101/SQB.1994.059.01.040 7587088

[55] Kallen AN Zhou X-B Xu J Qiao C Ma J Yan L. The imprinted H19 lncRNA antagonizes let-7 microRNAs. Mol Cell (2013) 52:101–12. 10.1016/j.molcel.2013.08.027 24055342 PMC3843377

[56] Leung A Trac C Jin W Lanting L Akbany A Sætrom P. Novel long noncoding RNAs are regulated by angiotensin II in vascular smooth muscle cells. Circ Res. (2013) 113:266–78. 10.1161/CIRCRESAHA.112.300849 23697773 PMC3763837

[57] Zhao J Zhang W Lin M Wu W Jiang P Tou E. MYOSLID is a novel serum response factor-dependent long noncoding rna that amplifies the vascular smooth muscle differentiation program. Arterioscler Thromb Vasc Biol. (2016) 36:2088–99. 10.1161/ATVBAHA.116.307879 27444199 PMC5033703

[58] Krawczyk M Emerson BM. p50-associated COX-2 extragenic RNA (PACER) activates COX-2 gene expression by occluding repressive NF-κB complexes. Elife (2014) 3:e01776. 10.7554/eLife.01776 24843008 PMC4017649

[59] Bell RD Long X Lin M Bergmann JH Nanda V Cowan SL. Identification and initial functional characterization of a human vascular cell-enriched long noncoding RNA. Arterioscler Thromb Vasc Biol. (2014) 34:1249–59. 10.1161/ATVBAHA.114.303240 24578380 PMC4024079

[60] Ballantyne MD Pinel K Dakin R Vesey AT Diver L Mackenzie R. Smooth muscle enriched long noncoding RNA (SMILR) regulates cell proliferation. Circulation (2016) 133:2050–65. 10.1161/CIRCULATIONAHA.115.021019 27052414 PMC4872641

[61] Singh KK Matkar PN Pan Y Quan A Gupta V Teoh H. Endothelial long non-coding RNAs regulated by oxidized LDL. Mol Cell Biochem. (2017) 431:139–49. 10.1007/s11010-017-2984-2 28316063

[62] Neumann P Jaé N Knau A Glaser SF Fouani Y Rossbach O. The lncRNA GATA6-AS epigenetically regulates endothelial gene expression via interaction with LOXL2. Nat Commun. (2018) 9:237. 10.1038/s41467-017-02431-1 29339785 PMC5770451

[63] Miao Y Ajami NE Huang T-S Lin F-M Lou C-H Wang Y-T. Enhancer-associated long non-coding RNA LEENE regulates endothelial nitric oxide synthase and endothelial function. Nat Commun. (2018) 9:292. 10.1038/s41467-017-02113-y 29348663 PMC5773557

[64] Ishii N Ozaki K Sato H Mizuno H Saito S Takahashi A. Identification of a novel non-coding RNA, MIAT, that confers risk of myocardial infarction. J Hum Genet. (2006) 51:1087–99. 10.1007/s10038-006-0070-9 17066261

[65] Yan B Yao J Liu J-Y Li X-M Wang X-Q Li Y-J. lncRNA-MIAT regulates microvascular dysfunction by functioning as a competing endogenous RNA. Circ Res. (2015) 116:1143–56. 10.1161/CIRCRESAHA.116.305510 25587098

[66] Willingham AT Orth AP Batalov S Peters EC Wen BG Aza-Blanc P. A strategy for probing the function of noncoding RNAs finds a repressor of NFAT. Science (2005) 309:1570–3. 10.1126/science.1115901 16141075

[67] Tang Y Wo L Chai H. [Effects of noncoding RNA NRON gene regulation on human umbilical vein endothelial cells functions]. Zhonghua Xin Xue Guan Bing Za Zhi. (2013) 41:245–50. 10.3760/cma.j.issn.0253-3758.2013.03.015 23879952

[68] Tsao H-W Tai T-S Tseng W Chang H-H Grenningloh R Miaw S-C. Ets-1 facilitates nuclear entry of NFAT proteins and their recruitment to the IL-2 promoter. Proc Natl Acad Sci USA. (2013) 110:15776–81. 10.1073/pnas.1304343110 24019486 PMC3785780

[69] Robb GB Carson AR Tai SC Fish JE Singh S Yamada T. Post-transcriptional regulation of endothelial nitric-oxide synthase by an overlapping antisense mRNA transcript. J Biol Chem. (2004) 279:37982–96. 10.1074/jbc.M400271200 15234981

[70] Man HSJ Sukumar AN Lam GC Turgeon PJ Yan MS Ku KH. Angiogenic patterning by STEEL, an endothelial-enriched long noncoding RNA. Proc Natl Acad Sci USA. (2018) 115:2401–6. 10.1073/pnas.1715182115 29467285 PMC5877935

[71] Li K Blum Y Verma A Liu Z Pramanik K Leigh NR. A noncoding antisense RNA in tie-1 locus regulates tie-1 function *in vivo*. Blood (2010) 115:133–9. 10.1182/blood-2009-09-242180 19880500 PMC2803688

[72] Chowdhury TA Koceja C Eisa-Beygi S Kleinstiver BP Kumar SN Lin C-W. Temporal and Spatial Post-Transcriptional Regulation of Zebrafish tie1 mRNA by long noncoding RNA during brain vascular assembly. Arterioscler Thromb Vasc Biol. (2018) 38:1562–75. 10.1161/ATVBAHA.118.310848 29724820 PMC6023729

[73] Das S Reddy MA Senapati P Stapleton K Lanting L Wang M. Diabetes mellitus-induced long noncoding RNA Dnm3os regulates macrophage functions and inflammation via nuclear mechanisms. Arterioscler Thromb Vasc Biol. (2018) 38:1806–20. 10.1161/ATVBAHA.117.310663 29930005 PMC6202204

[74] Loebel DAF Tsoi B Wong N Tam PPL. A conserved noncoding intronic transcript at the mouse Dnm3 locus. Genomics (2005) 85:782–9. 10.1016/j.ygeno.2005.02.001 15885504

[75] Du M Yuan L Tan X Huang D Wang X Zheng Z. The LPS-inducible lncRNA Mirt2 is a negative regulator of inflammation. Nat Commun. (2017) 8:2049. 10.1038/s41467-017-02229-1 29230038 PMC5725456

[76] Sallam T Jones M Thomas BJ Wu X Gilliland T Qian K. Transcriptional regulation of macrophage cholesterol efflux and atherogenesis by a long noncoding RNA. Nat Med. (2018) 24:304–12. 10.1038/nm.4479 29431742 PMC5839972

[77] Halley P Kadakkuzha BM Faghihi MA Magistri M Zeier Z Khorkova O. Regulation of the apolipoprotein gene cluster by a long noncoding RNA. Cell Rep. (2014) 6:222–30. 10.1016/j.celrep.2013.12.015 24388749 PMC3924898

[78] Sallam T Jones MC Gilliland T Zhang L Wu X Eskin A. Feedback modulation of cholesterol metabolism by the lipid-responsive non-coding RNA LeXis. Nature (2016) 534:124–8. 10.1038/nature17674 27251289 PMC4896091

[79] Li P Ruan X Yang L Kiesewetter K Zhao Y Luo H. A liver-enriched long non-coding RNA, lncLSTR, regulates systemic lipid metabolism in mice. Cell Metab. (2015) 21:455–67. 10.1016/j.cmet.2015.02.004 25738460 PMC4350020

[80] Douvris A Soubeyrand S Naing T Martinuk A Nikpay M Williams A. Functional analysis of the TRIB1 associated locus linked to plasma triglycerides and coronary artery disease. J Am Heart Assoc. (2014) 3:e000884. 10.1161/JAHA.114.000884 24895164 PMC4309087

[81] Congrains A Kamide K Ohishi M Rakugi H. ANRIL: molecular mechanisms and implications in human health. Int J Mol Sci. (2013) 14:1278–92. 10.3390/ijms14011278 23306151 PMC3565320

[82] Kotake Y Nakagawa T Kitagawa K Suzuki S Liu N Kitagawa M. Long non-coding RNA ANRIL is required for the PRC2 recruitment to and silencing of p15(INK4B) tumor suppressor gene. Oncogene (2011) 30:1956–62. 10.1038/onc.2010.568 21151178 PMC3230933

[83] Yap KL Li S Muñoz-Cabello AM Raguz S Zeng L Mujtaba S. Molecular interplay of the noncoding RNA ANRIL and methylated histone H3 lysine 27 by polycomb CBX7 in transcriptional silencing of INK4a. Mol Cell. (2010) 38:662–74. 10.1016/j.molcel.2010.03.021 20541999 PMC2886305

[84] Motterle A Pu X Wood H Xiao Q Gor S Ng FL. Functional analyses of coronary artery disease associated variation on chromosome 9p21 in vascular smooth muscle cells. Hum Mol Genet. (2012) 21:4021–9. 10.1093/hmg/dds224 22706276 PMC3428153

[85] Zhou X Han X Wittfeldt A Sun J Liu C Wang X. Long non-coding RNA ANRIL regulates inflammatory responses as a novel component of NF-κB pathway. RNA Biol. (2016) 13:98–108. 10.1080/15476286.2015.1122164 26618242 PMC4829310

[86] Visel A Zhu Y May D Afzal V Gong E Attanasio C. Targeted deletion of the 9p21 non-coding coronary artery disease risk interval in mice. Nature (2010) 464:409–12. 10.1038/nature08801 20173736 PMC2938076

[87] Holdt LM Teupser D. Long Noncoding RNA ANRIL: Lnc-ing genetic variation at the chromosome 9p21 locus to molecular mechanisms of atherosclerosis. Front Cardiovasc Med. (2018) 5:145. 10.3389/fcvm.2018.00145 30460243 PMC6232298

[88] Arslan S Berkan Ö Lalem T Özbilüm N Göksel S Korkmaz Ö. Long non-coding RNAs in the atherosclerotic plaque. Atherosclerosis (2017) 266:176–81. 10.1016/j.atherosclerosis.2017.10.012 29035780

[89] Cremer S Michalik KM Fischer A Pfisterer L Jaé N Winter C. Hematopoietic deficiency of the long non-coding RNA MALAT1 promotes atherosclerosis and plaque inflammation. Circulation (2018). [Epub ahead of print]. 10.1161/CIRCULATIONAHA.117.029015 30586743

[90] Koldemir O Özgür E Gezer U. Accumulation of GAS5 in exosomes is a marker of apoptosis induction. Biomed Rep. (2017) 6:358–62. 10.3892/br.2017.848 28451400 PMC5403406

[91] Aryal B Rotllan N Fernández-Hernando C. Noncoding RNAs and atherosclerosis. Curr Atheroscler Rep. (2014) 16:407. 10.1007/s11883-014-0407-3 24623179 PMC4145585

[92] Zhou T Ding J Wang X Zheng X. Long noncoding RNAs and atherosclerosis. Atherosclerosis (2016) 248:51–61. 10.1016/j.atherosclerosis.2016.02.025 26987066

[93] Gast M Rauch BH Nakagawa S Haghikia A Jasina A Haas J Immune system-mediated atherosclerosis caused by deficiency of long noncoding RNA MALAT1 in ApoE-/- mice. Cardiovasc Res. (2018) 115:302–14. 10.1093/cvr/cvy202 30101304

[94] Liu J Huang G-Q Ke Z-P Silence of long intergenic noncoding RNA HOTAIR ameliorates oxidative stress and inflammation response in ox-LDL-treated human macrophages by upregulating miR-330–5 p. J Cell Physiol. (2019) 234:5134–42. 10.1002/jcp.27317 30187491

[95] Soubeyrand S Naing T Martinuk A McPherson R. ERK1/2 regulates hepatocyte Trib1 in response to mitochondrial dysfunction. Biochim Biophys Acta. (2013) 1833:3405–14. 10.1016/j.bbamcr.2013.10.001 24161842

[96] Kiss-Toth E Bagstaff SM Sung HY Jozsa V Dempsey C Caunt JC. Human tribbles, a protein family controlling mitogen-activated protein kinase cascades. J Biol Chem. (2004) 279:42703–8. 10.1074/jbc.M407732200 15299019

[97] Hannou SA Wouters K Paumelle R Staels B. Functional genomics of the CDKN2A/B locus in cardiovascular and metabolic disease: what have we learned from GWASs? Trends Endocrinol Metab. (2015) 26:176–84. 10.1016/j.tem.2015.01.008 25744911

[98] Holdt LM Beutner F Scholz M Gielen S Gäbel G Bergert H. ANRIL expression is associated with atherosclerosis risk at chromosome 9p21. Arterioscler Thromb Vasc Biol. (2010) 30:620–7. 10.1161/ATVBAHA.109.196832 20056914

[99] Zhou J Xu J Zhang L Liu S Ma Y Wen X. Combined single-cell profiling of lncRNAs and functional screening reveals that H19 is pivotal for embryonic hematopoietic stem cell development. Cell Stem Cell. (2019) 24:285–98.e5. 10.1016/j.stem.2018.11.023 30639035

[100] Yoshimura H Matsuda Y Yamamoto M Kamiya S Ishiwata T. Expression and role of long non-coding RNA H19 in carcinogenesis. Front Biosci. (2018) 23:614–25. 10.2741/4608 28930564

[101] Gao W Zhu M Wang H Zhao S Zhao D Yang Y. Association of polymorphisms in long non-coding RNA H19 with coronary artery disease risk in a Chinese population. Mutat Res. (2015) 772:15–22. 10.1016/j.mrfmmm.2014.12.009 25772106

[102] Zhu R Liu X He Z. Long non-coding RNA H19 and MALAT1 gene variants in patients with ischemic stroke in a northern Chinese Han population. Mol Brain (2018) 11:58. 10.1186/s13041-018-0402-7 30305120 PMC6180423

[103] Kim DK Zhang L Dzau VJ Pratt RE. H19, a developmentally regulated gene, is reexpressed in rat vascular smooth muscle cells after injury. J Clin Invest. (1994) 93:355–60. 10.1172/JCI116967 8282806 PMC293782

[104] Sun W Lv J Duan L Lin R Li Y Li S. Long noncoding RNA H19 promotes vascular remodeling by sponging let-7a to upregulate the expression of cyclin D1. Biochem Biophys Res Commun. (2019) 508:1038–42. 10.1016/j.bbrc.2018.11.185 30551879

[105] Hofmann P Sommer J Theodorou K Kirchhof L Fischer A Li Y. Long non-coding RNA H19 regulates endothelial cell aging via inhibition of STAT3 signalling. Cardiovasc Res. (2019) 115:230–42. 10.1093/cvr/cvy206 30107531 PMC6302267

[106] Boon RA Hofmann P Michalik KM Lozano-Vidal N Berghäuser D Fischer A. Long noncoding RNA meg3 controls endothelial cell aging and function: implications for regenerative angiogenesis. J Am Coll Cardiol. (2016) 68:2589–91. 10.1016/j.jacc.2016.09.949 27931619

[107] van der Harst P Verweij N. Identification of 64 novel genetic loci provides an expanded view on the genetic architecture of coronary artery disease. Circ Res. (2018) 122:433–43. 10.1161/CIRCRESAHA.117.312086 29212778 PMC5805277

[108] Liu B Pjanic M Wang T Nguyen T Gloudemans M Rao A. Genetic regulatory mechanisms of smooth muscle cells map to coronary artery disease risk loci. Am J Hum Genet. (2018) 103:377–88. 10.1016/j.ajhg.2018.08.001 30146127 PMC6128252

[109] Arab K Park YJ Lindroth AM Schäfer A Oakes C Weichenhan D. Long noncoding RNA TARID directs demethylation and activation of the tumor suppressor TCF21 via GADD45A. Mol Cell. (2014) 55:604–14. 10.1016/j.molcel.2014.06.031 25087872

[110] Acharya A Baek ST Huang G Eskiocak B Goetsch S Sung CY. The bHLH transcription factor Tcf21 is required for lineage-specific EMT of cardiac fibroblast progenitors. Development (2012) 139:2139–49. 10.1242/dev.079970 22573622 PMC3357908

[111] Miller CL Anderson DR Kundu RK Raiesdana A Nürnberg ST Diaz R. Disease-related growth factor and embryonic signaling pathways modulate an enhancer of TCF21 expression at the 6q23.2 coronary heart disease locus. PLoS Genet. (2013) 9:e1003652. 10.1371/journal.pgen.1003652 23874238 PMC3715442

[112] Miller CL Haas U Diaz R Leeper NJ Kundu RK Patlolla B. Coronary heart disease-associated variation in TCF21 disrupts a miR-224 binding site and miRNA-mediated regulation. PLoS Genet. (2014) 10:e1004263. 10.1371/journal.pgen.1004263 24676100 PMC3967965

[113] Nurnberg ST Cheng K Raiesdana A Kundu R Miller CL Kim JB. Coronary artery disease associated transcription factor TCF21 regulates smooth muscle precursor cells that contribute to the fibrous cap. PLoS Genet. (2015) 11:e1005155. 10.1371/journal.pgen.1005155 26020946 PMC4447275

[114] Hormozdiari F van de Bunt M Segrè AV Li X Joo JWJ Bilow M. Colocalization of GWAS and eQTL signals detects target genes. Am J Hum Genet. (2016) 99:1245–60. 10.1016/j.ajhg.2016.10.003 27866706 PMC5142122

[115] Gusev A Ko A Shi H Bhatia G Chung W Penninx BWJH. Integrative approaches for large-scale transcriptome-wide association studies. Nat Genet. (2016) 48:245–52. 10.1038/ng.3506 26854917 PMC4767558

[116] Kashi K Henderson L Bonetti A Carninci P. Discovery and functional analysis of lncRNAs: Methodologies to investigate an uncharacterized transcriptome. Biochim Biophys Acta. (2016) 1859:3–15. 10.1016/j.bbagrm.2015.10.010 26477492

[117] Kukurba KR Montgomery SB. RNA sequencing and analysis. Cold Spring Harb Protoc. (2015) 2015:951–69. 10.1101/pdb.top084970 25870306 PMC4863231

[118] Freedman JE Miano JM, National Heart, Lung, and Blood Institute Workshop Participants^*^. Challenges and opportunities in linking long noncoding rnas to cardiovascular, lung, and blood diseases. Arterioscler Thromb Vasc Biol. (2017) 37:21–5. 10.1161/ATVBAHA.116.308513 27856459 PMC5222717

[119] Derrien T Johnson R Bussotti G Tanzer A Djebali S Tilgner H. The GENCODE v7 catalog of human long noncoding RNAs: analysis of their gene structure, evolution, and expression. Genome Res. (2012) 22:1775–89. 10.1101/gr.132159.111 22955988 PMC3431493

[120] Conesa A Madrigal P Tarazona S Gomez-Cabrero D Cervera A McPherson A A survey of best practices for RNA-seq data analysis. Genome Biol. (2016) 17:13 10.1186/s13059-016-0881-8 26813401 PMC4728800

[121] Cabili MN Trapnell C Goff L Koziol M Tazon-Vega B Regev A. Integrative annotation of human large intergenic noncoding RNAs reveals global properties and specific subclasses. Genes Dev. (2011) 25:1915–27. 10.1101/gad.17446611 21890647 PMC3185964

[122] Guil S Soler M Portela A Carrère J Fonalleras E Gómez A. Intronic RNAs mediate EZH2 regulation of epigenetic targets. Nat Struct Mol Biol. (2012) 19:664–70. 10.1038/nsmb.2315 22659877

[123] Tsoi LC Iyer MK Stuart PE Swindell WR Gudjonsson JE Tejasvi T. Analysis of long non-coding RNAs highlights tissue-specific expression patterns and epigenetic profiles in normal and psoriatic skin. Genome Biol. (2015) 16:24. 10.1186/s13059-014-0570-4 25723451 PMC4311508

[124] Ackers-Johnson M Talasila A Sage AP Long X Bot I Morrell NW. Myocardin regulates vascular smooth muscle cell inflammatory activation and disease. Arterioscler Thromb Vasc Biol. (2015) 35:817–28. 10.1161/ATVBAHA.114.305218 25614278 PMC4390125

[125] Chen J Kitchen CM Streb JW Miano JM. Myocardin: a component of a molecular switch for smooth muscle differentiation. J Mol Cell Cardiol. (2002) 34:1345–56. 10.1006/jmcc.2002.2086 12392995

[126] Wang D-Z Li S Hockemeyer D Sutherland L Wang Z Schratt G. Potentiation of serum response factor activity by a family of myocardin-related transcription factors. Proc Natl Acad Sci USA. (2002) 99:14855–60. 10.1073/pnas.222561499 12397177 PMC137508

[127] Du KL Ip HS Li J Chen M Dandre F Yu W. Myocardin is a critical serum response factor cofactor in the transcriptional program regulating smooth muscle cell differentiation. Mol Cell Biol. (2003) 23:2425–37. 10.1128/MCB.23.7.2425-2437.2003 12640126 PMC150745

[128] Yoshida T Sinha S Dandré F Wamhoff BR Hoofnagle MH Kremer BE. Myocardin is a key regulator of CArG-dependent transcription of multiple smooth muscle marker genes. Circ Res. (2003) 92:856–64. 10.1161/01.RES.0000068405.49081.09 12663482

[129] Russell MR Penikis A Oldridge DA Alvarez-Dominguez JR McDaniel L Diamond M. CASC15-S is a tumor suppressor lncRNA at the 6p22 neuroblastoma susceptibility locus. Cancer Res. (2015) 75:3155–66. 10.1158/0008-5472.CAN-14-3613 26100672 PMC4526355

[130] Lim Y-H Kwon D-H Kim J Park WJ Kook H Kim Y-K. Identification of long noncoding RNAs involved in muscle differentiation. PLoS ONE (2018) 13:e0193898. 10.1371/journal.pone.0193898 29499054 PMC5834194

[131] Mantella L-E Singh KK Sandhu P Kantores C Ramadan A Khyzha N. Fingerprint of long non-coding RNA regulated by cyclic mechanical stretch in human aortic smooth muscle cells: implications for hypertension. Mol Cell Biochem. (2017) 435(1–2):163–73. 10.1007/s11010-017-3065-2 28526936

[132] Zhang X Tang X Hamblin MH Yin K-J. Long Non-Coding RNA Malat1 regulates angiogenesis in Hindlimb Ischemia. Int J Mol Sci. (2018) 19:E1723. 10.3390/ijms19061723 29891768 PMC6032369

[133] Huang C Han J Wu Y Li S Wang Q Lin W. Exosomal MALAT1 derived from oxidized low-density lipoprotein-treated endothelial cells promotes M2 macrophage polarization. Mol Med Report. (2018) 18:509–15. 10.3892/mmr.2018.8982 29750307

[134] He C Yang W Yang J Ding J Li S Wu H. Long noncoding RNA MEG3 negatively regulates proliferation and angiogenesis in vascular endothelial cells. DNA Cell Biol. (2017) 36:475–81. 10.1089/dna.2017.3682 28418724

[135] Liu J Li Q Zhang K-S Hu B Niu X Zhou S-M. Downregulation of the Long Non-Coding RNA Meg3 Promotes Angiogenesis After Ischemic Brain Injury by Activating Notch Signaling. Mol Neurobiol. (2017) 54:8179–90. 10.1007/s12035-016-0270-z 27900677 PMC5684256

[136] Boulberdaa M Scott E Ballantyne M Garcia R Descamps B Angelini GD. A role for the long noncoding RNA SENCR in commitment and function of endothelial cells. Mol Ther. (2016) 24:978–90. 10.1038/mt.2016.41 26898221 PMC4876031

[137] Zhang H Xue C Wang Y Shi J Zhang X Li W. Deep RNA sequencing uncovers a repertoire of human macrophage long intergenic noncoding rnas modulated by macrophage activation and associated with cardiometabolic diseases. J Am Heart Assoc. (2017) 6:e007431. 10.1161/JAHA.117.007431 29133519 PMC5721798

[138] Xue C Zhang X Zhang H Ferguson JF Wang Y Hinkle CC. De novo RNA sequence assembly during in vivo inflammatory stress reveals hundreds of unannotated lincRNAs in human blood CD14+ monocytes and in adipose tissue. Physiol Genomics (2017) 49:287–305. 10.1152/physiolgenomics.00001.2017 28389524 PMC5495909

[139] Hong C Tontonoz P. Liver X receptors in lipid metabolism: opportunities for drug discovery. Nat Rev Drug Discov. (2014) 13:433–44. 10.1038/nrd4280 24833295

[140] Parikh M Patel K Soni S Gandhi T. Liver X receptor: a cardinal target for atherosclerosis and beyond. J Atheroscler Thromb. (2014) 21:519–31. 10.5551/jat.19778 24695022

[141] Zhang Y Breevoort SR Angdisen J Fu M Schmidt DR Holmstrom SR. Liver LXRα expression is crucial for whole body cholesterol homeostasis and reverse cholesterol transport in mice. J Clin Invest. (2012) 122:1688–99. 10.1172/JCI59817 22484817 PMC3336978

[142] Tontonoz P Wu X Jones M Zhang Z Salisbury D Sallam T. Long noncoding RNA facilitated gene therapy reduces atherosclerosis in a murine model of familial hypercholesterolemia. Circulation (2017) 136:776–8. 10.1161/CIRCULATIONAHA.117.029002 28827223 PMC5657526

[143] Tilgner H Jahanbani F Blauwkamp T Moshrefi A Jaeger E Chen F. Comprehensive transcriptome analysis using synthetic long-read sequencing reveals molecular co-association of distant splicing events. Nat Biotechnol. (2015) 33:736–42. 10.1038/nbt.3242 25985263 PMC4832928

[144] Uszczynska-Ratajczak B Lagarde J Frankish A Guigó R Johnson R. Towards a complete map of the human long non-coding RNA transcriptome. Nat Rev Genet. (2018) 19:535–48. 10.1038/s41576-018-0017-y 29795125 PMC6451964

[145] Jain M Olsen HE Paten B Akeson M The Oxford Nanopore MinION: delivery of nanopore sequencing to the genomics community. Genome Biol. (2016) 17:239 10.1186/s13059-016-1103-0 27887629 PMC5124260

[146] Lagarde J Uszczynska-Ratajczak B Santoyo-Lopez J Gonzalez JM Tapanari E Mudge JM. Extension of human lncRNA transcripts by RACE coupled with long-read high-throughput sequencing (RACE-Seq). Nat Commun. (2016) 7:12339. 10.1038/ncomms12339 27531712 PMC4992054

[147] Mercer TR Clark MB Crawford J Brunck ME Gerhardt DJ Taft RJ. Targeted sequencing for gene discovery and quantification using RNA CaptureSeq. Nat Protoc. (2014) 9:989–1009. 10.1038/nprot.2014.058 24705597

[148] Clark MB Mercer TR Bussotti G Leonardi T Haynes KR Crawford J. Quantitative gene profiling of long noncoding RNAs with targeted RNA sequencing. Nat Methods (2015) 12:339–42. 10.1038/nmeth.3321 25751143

[149] Bussotti G Leonardi T Clark MB Mercer TR Crawford J Malquori L. Improved definition of the mouse transcriptome via targeted RNA sequencing. Genome Res. (2016) 26:705–16. 10.1101/gr.199760.115 27197243 PMC4864457

[150] Lagarde J Uszczynska-Ratajczak B Carbonell S Pérez-Lluch S Abad A Davis C. High-throughput annotation of full-length long noncoding RNAs with capture long-read sequencing. Nat Genet. (2017) 49:1731–40. 10.1038/ng.3988 29106417 PMC5709232

[151] Chu C Quinn J Chang HY Chromatin isolation by RNA purification (ChIRP). J Vis Exp. (2012) e3912 10.3791/3912 PMC3460573 22472705

[152] Chu C Qu K Zhong FL Artandi SE Chang HY. Genomic maps of long noncoding RNA occupancy reveal principles of RNA-chromatin interactions. Mol Cell (2011) 44:667–78. 10.1016/j.molcel.2011.08.027 21963238 PMC3249421

[153] Quinn JJ Ilik IA Qu K Georgiev P Chu C Akhtar A. Revealing long noncoding RNA architecture and functions using domain-specific chromatin isolation by RNA purification. Nat Biotechnol. (2014) 32:933–40. 10.1038/nbt.2943 24997788 PMC4175979

[154] Simon MD Wang CI Kharchenko PV West JA Chapman BA Alekseyenko AA. The genomic binding sites of a noncoding RNA. Proc Natl Acad Sci USA. (2011) 108:20497–502. 10.1073/pnas.1113536108 22143764 PMC3251105

[155] Simon MD. Capture hybridization analysis of RNA targets (CHART). Curr Protoc Mol Biol. (2013) Chapter 21:Unit 21.25. 10.1002/0471142727.mb2125s101 23288463

[156] Li X Zhou B Chen L Gou L-T Li H Fu X-D. GRID-seq reveals the global RNA-chromatin interactome. Nat Biotechnol. (2017) 35:940–50. 10.1038/nbt.3968 28922346 PMC5953555

[157] Sridhar B Rivas-Astroza M Nguyen TC Chen W Yan Z Cao X Systematic Mapping of RNA-chromatin interactions *in vivo*. Curr Biol. (2017) 27:602–9. 10.1016/j.cub.2017.01.011 28132817 PMC5319903

[158] Chu C Zhang QC da Rocha ST Flynn RA Bharadwaj M Calabrese JM. Systematic discovery of Xist RNA binding proteins. Cell (2015) 161:404–16. 10.1016/j.cell.2015.03.025 25843628 PMC4425988

[159] Yang L Lin C Liu W Zhang J Ohgi KA Grinstein JD. ncRNA- and Pc2 methylation-dependent gene relocation between nuclear structures mediates gene activation programs. Cell (2011) 147:773–88. 10.1016/j.cell.2011.08.054 22078878 PMC3297197

[160] Chen R Liu Y Zhuang H Yang B Hei K Xiao M. Quantitative proteomics reveals that long non-coding RNA MALAT1 interacts with DBC1 to regulate p53 acetylation. Nucleic Acids Res. (2017) 45:9947–59. 10.1093/nar/gkx600 28973437 PMC5622371

[161] McHugh CA Guttman M. RAP-MS: a method to identify proteins that interact directly with a specific RNA molecule in cells. Methods Mol Biol. (2018) 1649:473–88. 10.1007/978-1-4939-7213-5_31 29130217

[162] Huppertz I Attig J D'Ambrogio A Easton LE Sibley CR Sugimoto Y. iCLIP: protein-RNA interactions at nucleotide resolution. Methods (2014) 65:274–87. 10.1016/j.ymeth.2013.10.011 24184352 PMC3988997

[163] Hafner M Landthaler M Burger L Khorshid M Hausser J Berninger P. Transcriptome-wide identification of RNA-binding protein and microRNA target sites by PAR-CLIP. Cell (2010) 141:129–41. 10.1016/j.cell.2010.03.009 20371350 PMC2861495

[164] Licatalosi DD Mele A Fak JJ Ule J Kayikci M Chi SW. HITS-CLIP yields genome-wide insights into brain alternative RNA processing. Nature (2008) 456:464–9. 10.1038/nature07488 18978773 PMC2597294

[165] Zarnegar BJ Flynn RA Shen Y Do BT Chang HY Khavari PA. irCLIP platform for efficient characterization of protein-RNA interactions. Nat Methods (2016) 13:489–92. 10.1038/nmeth.3840 27111506 PMC5477425

[166] Van Nostrand EL Pratt GA Shishkin AA Gelboin-Burkhart C Fang MY Sundararaman B. Robust transcriptome-wide discovery of RNA-binding protein binding sites with enhanced CLIP (eCLIP). Nat Methods (2016) 13:508–14. 10.1038/nmeth.3810 27018577 PMC4887338

[167] McMahon AC Rahman R Jin H Shen JL Fieldsend A Luo W. TRIBE: Hijacking an RNA-editing enzyme to identify cell-specific targets of RNA-binding proteins. Cell (2016) 165:742–53. 10.1016/j.cell.2016.03.007 27040499 PMC5027142

[168] Rahman R Xu W Jin H Rosbash M. Identification of RNA-binding protein targets with HyperTRIBE. Nat Protoc. (2018) 13:1829–49. 10.1038/s41596-018-0020-y 30013039 PMC6349038

[169] Engreitz J Lander ES Guttman M. RNA antisense purification (RAP) for mapping RNA interactions with chromatin. Methods Mol Biol. (2015) 1262:183–97. 10.1007/978-1-4939-2253-6_11 25555582

[170] Sharma E Sterne-Weiler T O'Hanlon D Blencowe BJ. Global mapping of human RNA-RNA Interactions. Mol Cell. (2016) 62:618–26. 10.1016/j.molcel.2016.04.030 27184080

[171] Lu Z Zhang QC Lee B Flynn RA Smith MA Robinson JT. RNA duplex map in living cells reveals higher-order transcriptome structure. Cell (2016) 165:1267–79. 10.1016/j.cell.2016.04.028 27180905 PMC5029792

[172] Aw JGA Shen Y Wilm A Sun M Lim XN Boon K-L. *In vivo* mapping of Eukaryotic RNA interactomes reveals principles of higher-order organization and regulation. Mol Cell. (2016) 62:603–17. 10.1016/j.molcel.2016.04.028 27184079

[173] Nguyen TC Cao X Yu P Xiao S Lu J Biase FH. Mapping RNA-RNA interactome and RNA structure *in vivo* by MARIO. Nat Commun. (2016) 7:12023. 10.1038/ncomms12023 27338251 PMC4931010

[174] Femino AM Fay FS Fogarty K Singer RH. Visualization of single RNA transcripts *in situ*. Science (1998) 280:585–90. 10.1126/science.280.5363.585 9554849

[175] Shah S Lubeck E Zhou W Cai L. seqFISH accurately detects transcripts in single cells and reveals robust spatial organization in the hippocampus. Neuron (2017) 94:752–758.e1. 10.1016/j.neuron.2017.05.008 28521130

[176] Lubeck E Coskun AF Zhiyentayev T Ahmad M Cai L. Single-cell *in situ* RNA profiling by sequential hybridization. Nat Methods (2014) 11:360–1. 10.1038/nmeth.2892 24681720 PMC4085791

[177] Moffitt JR Zhuang X. RNA Imaging with Multiplexed Error-Robust Fluorescence *in situ* Hybridization (MERFISH). Meth Enzymol. (2016) 572:1–49. 10.1016/bs.mie.2016.03.020 27241748 PMC5023431

[178] Moffitt JR Hao J Bambah-Mukku D Lu T Dulac C Zhuang X. High-performance multiplexed fluorescence *in situ* hybridization in culture and tissue with matrix imprinting and clearing. Proc Natl Acad Sci USA. (2016) 113:14456–61. 10.1073/pnas.1617699113 27911841 PMC5167177

[179] Eng C-HL Shah S Thomassie J Cai L. Profiling the transcriptome with RNA SPOTs. Nat Methods (2017) 14:1153–5. 10.1038/nmeth.4500 29131163 PMC5819366

[180] Ståhl PL Salmén F Vickovic S Lundmark A Navarro JF Magnusson J. Visualization and analysis of gene expression in tissue sections by spatial transcriptomics. Science (2016) 353:78–82. 10.1126/science.aaf2403 27365449

[181] Asp M Salmén F Ståhl PL Vickovic S Felldin U Löfling M. Spatial detection of fetal marker genes expressed at low level in adult human heart tissue. Sci Rep. (2017) 7:12941. 10.1038/s41598-017-13462-5 29021611 PMC5636908

[182] Wang J Zhao Y Zhou X Hiebert SW Liu Q Shyr Y. Nascent RNA sequencing analysis provides insights into enhancer-mediated gene regulation. BMC Genomics (2018) 19:633. 10.1186/s12864-018-5016-z 30139328 PMC6107967

[183] Tani H Mizutani R Salam KA Tano K Ijiri K Wakamatsu A. Genome-wide determination of RNA stability reveals hundreds of short-lived noncoding transcripts in mammals. Genome Res. (2012) 22:947–56. 10.1101/gr.130559.111 22369889 PMC3337439

[184] Sakurai M Ueda H Yano T Okada S Terajima H Mitsuyama T. A biochemical landscape of A-to-I RNA editing in the human brain transcriptome. Genome Res. (2014) 24:522–34. 10.1101/gr.162537.113 24407955 PMC3941116

[185] Nishikura K. A-to-I editing of coding and non-coding RNAs by ADARs. Nat Rev Mol Cell Biol. (2016) 17:83–96. 10.1038/nrm.2015.4 26648264 PMC4824625

[186] Nigita G Veneziano D Ferro A. A-to-I RNA editing: current knowledge sources and computational approaches with special emphasis on non-coding RNA molecules. Front Bioeng Biotechnol. (2015) 3:37. 10.3389/fbioe.2015.00037 25859542 PMC4373398

[187] Stellos K Gatsiou A Stamatelopoulos K Perisic Matic L John D Lunella FF. Adenosine-to-inosine RNA editing controls cathepsin S expression in atherosclerosis by enabling HuR-mediated post-transcriptional regulation. Nat Med. (2016) 22:1140–50. 10.1038/nm.4172 27595325

[188] Guttman M Rinn JL. Modular regulatory principles of large non-coding RNAs. Nature (2012) 482:339–46. 10.1038/nature10887 22337053 PMC4197003

[189] Schlackow M Nojima T Gomes T Dhir A Carmo-Fonseca M Proudfoot NJ. Distinctive patterns of transcription and RNA processing for human lincRNAs. Mol Cell (2017) 65:25–38. 10.1016/j.molcel.2016.11.029 28017589 PMC5222723

[190] Steijger T Abril JF Engström PG Kokocinski F RGASP Consortium Hubbard TJ. Assessment of transcript reconstruction methods for RNA-seq. Nat Methods (2013) 10:1177–84. 10.1038/nmeth.2714 24185837 PMC3851240

[191] You B-H Yoon S-H Nam J-W. High-confidence coding and noncoding transcriptome maps. Genome Res. (2017) 27:1050–62. 10.1101/gr.214288.116 28396519 PMC5453319

[192] Fu S Ma Y Yao H Xu Z Chen S Song J. IDP-denovo: *de novo* transcriptome assembly and isoform annotation by hybrid sequencing. Bioinformatics (2018) 34:2168–76. 10.1093/bioinformatics/bty098 29905763 PMC6022631

[193] Harrow J Frankish A Gonzalez JM Tapanari E Diekhans M Kokocinski F. GENCODE: the reference human genome annotation for The ENCODE Project. Genome Res. (2012) 22:1760–74. 10.1101/gr.135350.111 22955987 PMC3431492

[194] O'Leary NA Wright MW Brister JR Ciufo S Haddad D McVeigh R. Reference sequence (RefSeq) database at NCBI: current status, taxonomic expansion, and functional annotation. Nucleic Acids Res. (2016) 44(D1):D733–45. 10.1093/nar/gkv1189 26553804 PMC4702849

[195] Fang S Zhang L Guo J Niu Y Wu Y Li H. NONCODEV5: a comprehensive annotation database for long non-coding RNAs. Nucleic Acids Res. (2018) 46(D1):D308–14. 10.1093/nar/gkx1107 29140524 PMC5753287

[196] Hou M Tang X Tian F Shi F Liu F Gao G. AnnoLnc: a web server for systematically annotating novel human lncRNAs. BMC Genomics (2016) 17:931. 10.1186/s12864-016-3287-9 27852242 PMC5112684

[197] Wucher V Legeai F Hédan B Rizk G Lagoutte L Leeb T. FEELnc: a tool for long non-coding RNA annotation and its application to the dog transcriptome. Nucleic Acids Res. (2017) 45:e57. 10.1093/nar/gkw1306 28053114 PMC5416892

[198] Yang C Yang L Zhou M Xie H Zhang C Wang MD. LncADeep: an ab initio lncRNA identification and functional annotation tool based on deep learning. Bioinformatics (2018) 34:3825–34. 10.1093/bioinformatics/bty428 29850816

[199] Zhou J Huang Y Ding Y Yuan J Wang H Sun H. lncFunTK: a toolkit for functional annotation of long noncoding RNAs. Bioinformatics (2018) 34:3415–6. 10.1093/bioinformatics/bty339 29718162

[200] Cao Z Pan X Yang Y Huang Y Shen H-B. The lncLocator: a subcellular localization predictor for long non-coding RNAs based on a stacked ensemble classifier. Bioinformatics (2018) 34:2185–94. 10.1093/bioinformatics/bty085 29462250

[201] Hu Y Lin J Hu J Hu G Wang K Zhang H. PennDiff: detecting differential alternative splicing and transcription by RNA sequencing. Bioinformatics (2018) 34:2384–91. 10.1093/bioinformatics/bty097 29474557 PMC6041879

[202] Kirk JM Kim SO Inoue K Smola MJ Lee DM Schertzer MD. Functional classification of long non-coding RNAs by k-mer content. Nat Genet. (2018) 50:1474–82. 10.1038/s41588-018-0207-8 30224646 PMC6262761

[203] Sun Z Nair A Chen X Prodduturi N Wang J Kocher J-P. UClncR: Ultrafast and comprehensive long non-coding RNA detection from RNA-seq. Sci Rep. (2017) 7:14196. 10.1038/s41598-017-14595-3 29079769 PMC5660178

[204] Schneider HW Raiol T Brigido MM Walter MEMT Stadler PF. A support vector machine based method to distinguish long non-coding RNAs from protein coding transcripts. BMC Genomics (2017) 18:804. 10.1186/s12864-017-4178-4 29047334 PMC5648457

[205] Zhao J Song X Wang K. lncScore: alignment-free identification of long noncoding RNA from assembled novel transcripts. Sci Rep. (2016) 6:34838. 10.1038/srep34838 27708423 PMC5052565

[206] Hu L Xu Z Hu B Lu ZJ. COME: a robust coding potential calculation tool for lncRNA identification and characterization based on multiple features. Nucleic Acids Res. (2017) 45:e2. 10.1093/nar/gkw798 27608726 PMC5224497

[207] Sun L Liu H Zhang L Meng J. lncRScan-SVM: a tool for predicting long non-coding RNAs using support vector machine. PLoS ONE (2015) 10:e0139654. 10.1371/journal.pone.0139654 26437338 PMC4593643

[208] Baek J Lee B Kwon S Yoon S. lncRNAnet: long Non-coding RNA identification using deep learning. Bioinformatics (2018) 34:3889–97. 10.1093/bioinformatics/bty418 29850775

[209] Fan X-N Zhang S-W. lncRNA-MFDL: identification of human long non-coding RNAs by fusing multiple features and using deep learning. Mol Biosyst. (2015) 11:892–7. 10.1039/C4MB00650J 25588719

[210] Kang Y-J Yang D-C Kong L Hou M Meng Y-Q Wei L. CPC2: a fast and accurate coding potential calculator based on sequence intrinsic features. Nucleic Acids Res. (2017) 45(W1):W12–6. 10.1093/nar/gkx428 28521017 PMC5793834

[211] Kanehisa M Goto S Furumichi M Tanabe M Hirakawa M. KEGG for representation and analysis of molecular networks involving diseases and drugs. Nucleic Acids Res. (2010) 38(Database issue):D355–60. 10.1093/nar/gkp896 19880382 PMC2808910

[212] Budach S Marsico A. pysster: classification of biological sequences by learning sequence and structure motifs with convolutional neural networks. Bioinformatics (2018) 34:3035–7. 10.1093/bioinformatics/bty222 29659719 PMC6129303

[213] Lorenz R Bernhart SH Höner Zu Siederdissen C Tafer H Flamm C Stadler PF. ViennaRNA Package 2.0. Algorithms Mol Biol. (2011) 6:26. 10.1186/1748-7188-6-26 22115189 PMC3319429

[214] Reuter JS Mathews DH. RNAstructure: software for RNA secondary structure prediction and analysis. BMC Bioinformatics (2010) 11:129. 10.1186/1471-2105-11-129 20230624 PMC2984261

[215] Markham NR Zuker M. UNAFold: software for nucleic acid folding and hybridization. Methods Mol Biol. (2008) 453:3–31. 10.1007/978-1-60327-429-6_1 18712296

[216] Pedersen JS Bejerano G Siepel A Rosenbloom K Lindblad-Toh K Lander ES. Identification and classification of conserved RNA secondary structures in the human genome. PLoS Comput Biol. (2006) 2:e33. 10.1371/journal.pcbi.0020033 16628248 PMC1440920

[217] Nelson ADL Devisetty UK Palos K Haug-Baltzell AK Lyons E Beilstein MA. Evolinc: a tool for the identification and evolutionary comparison of long intergenic non-coding RNAs. Front Genet. (2017) 8:52. 10.3389/fgene.2017.00052 28536600 PMC5422434

[218] Gruber AR Neuböck R Hofacker IL Washietl S. The RNAz web server: prediction of thermodynamically stable and evolutionarily conserved RNA structures. Nucleic Acids Res. (2007) 35(Web Server issue):W335–8. 10.1093/nar/gkm222 17452347 PMC1933143

[219] Gesell T Washietl S. Dinucleotide controlled null models for comparative RNA gene prediction. BMC Bioinformatics (2008) 9:248. 10.1186/1471-2105-9-248 18505553 PMC2453142

[220] Talyan S Andrade-Navarro MA Muro EM. Identification of transcribed protein coding sequence remnants within lincRNAs. Nucleic Acids Res. (2018) 46:8720–9. 10.1093/nar/gky608 29986053 PMC6158594

[221] Zhang X Xue C Lin J Ferguson JF Weiner A Liu W. Interrogation of nonconserved human adipose lincRNAs identifies a regulatory role of linc-ADAL in adipocyte metabolism. Sci Transl Med. (2018) 10:aar5987. 10.1126/scitranslmed.aar5987 29925637 PMC6620026

[222] Aparicio-Prat E Arnan C Sala I Bosch N Guigo R Johnson R. DECKO: Single-oligo, dual-CRISPR deletion of genomic elements including long non-coding RNAs. BMC Genomics (2015) 16:846. 10.1186/s12864-015-2086-z 26493208 PMC4619085

[223] Covarrubias S Robinson EK Shapleigh B Vollmers A Katzman S Hanley N CRISPR/Cas-based screening of long non-coding RNAs (lncRNAs) in macrophages with an NF-KB reporter. J Biol Chem. (2017) 292:20911–20. 10.1074/jbc.M117.799155 29051223 PMC5743067

[224] Kim DH Marinov GK Pepke S Singer ZS He P Williams B. Single-cell transcriptome analysis reveals dynamic changes in lncRNA expression during reprogramming. Cell Stem Cell. (2015) 16:88–101. 10.1016/j.stem.2014.11.005 25575081 PMC4291542

[225] Liu SJ Nowakowski TJ Pollen AA Lui JH Horlbeck MA Attenello FJ. Single-cell analysis of long non-coding RNAs in the developing human neocortex. Genome Biol. (2016) 17:67. 10.1186/s13059-016-0932-1 27081004 PMC4831157

